# Biotechnological synthesis of Pd/Ag and Pd/Au nanoparticles for enhanced Suzuki–Miyaura cross‐coupling activity

**DOI:** 10.1111/1751-7915.13762

**Published:** 2021-03-15

**Authors:** Richard L. Kimber, Fabio Parmeggiani, Thomas S. Neill, Mohamed L. Merroun, Gregory Goodlet, Nigel A. Powell, Nicholas J. Turner, Jonathan R. Lloyd

**Affiliations:** ^1^ Department of Earth and Environmental Sciences and Williamson Research Centre for Molecular Environmental Science University of Manchester Manchester UK; ^2^ Department of Chemistry Manchester Institute of Biotechnology (MIB) University of Manchester Manchester UK; ^3^ Department of Microbiology Faculty of Sciences University of Granada Campus Fuentenueva Granada 18071 Spain; ^4^ Johnson Matthey Technology Centre Reading RG4 9NH UK; ^5^ Present address: Department of Environmental Geosciences University of Vienna Althanstraße 14 (UZA II) Vienna 1090 Austria; ^6^ Present address: Department of Chemistry, Materials and Chemical Engineering ‘G. Natta’ Politecnico di Milano Via Mancinelli 7 Milano 20131 Italy; ^7^ Present address: Institute for Nuclear Waste Disposal Karlsruhe Institute of Technology Karlsruhe 76021 Germany

## Abstract

Bimetallic nanoparticle catalysts have attracted considerable attention due to their unique chemical and physical properties. The ability of metal‐reducing bacteria to produce highly catalytically active monometallic nanoparticles is well known; however, the properties and catalytic activity of bimetallic nanoparticles synthesized with these organisms is not well understood. Here, we report the one‐pot biosynthesis of Pd/Ag (bio‐Pd/Ag) and Pd/Au (bio‐Pd/Au) nanoparticles using the metal‐reducing bacterium, *Shewanella oneidensis*, under mild conditions. Energy dispersive X‐ray analyses performed using scanning transmission electron microscopy (STEM) revealed the presence of both metals (Pd/Ag or Pd/Au) in the biosynthesized nanoparticles. X‐ray absorption near‐edge spectroscopy (XANES) suggested a significant contribution from Pd(0) and Pd(II) in both bio‐Pd/Ag and bio‐Pd/Au, with Ag and Au existing predominately as their metallic forms. Extended X‐ray absorption fine‐structure spectroscopy (EXAFS) supported the presence of multiple Pd species in bio‐Pd/Ag and bio‐Pd/Au, as inferred from Pd–Pd, Pd–O and Pd–S shells. Both bio‐Pd/Ag and bio‐Pd/Au demonstrated greatly enhanced catalytic activity towards Suzuki–Miyaura cross‐coupling compared to a monometallic Pd catalyst, with bio‐Pd/Ag significantly outperforming the others. The catalysts were very versatile, tolerating a wide range of substituents. This work demonstrates a green synthesis method for novel bimetallic nanoparticles that display significantly enhanced catalytic activity compared to their monometallic counterparts.

## Graphical abstract

This article reports the biotechnological synthesis of novel bimetallic nanoparticles which display significantly enhanced catalytic activity in Suzuki–Miyaura couplings, compared to their monometallic counterparts. In particular, the biosynthesised Pd/Ag was found to be very versatile, tolerating a range of substituents and generating products with a good to excellent yields, including pharmaceutically relevant compounds.

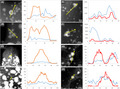

## Introduction

Nanoparticles make excellent catalysts due their high surface area to volume ratio and presence of defect sites (Bell, 2003; Kortlever *et al*., 2015). The synthesis and application of bimetallic nanoparticles has attracted considerable attention due to the unique catalytic properties arising from synergistic interactions between the two metal components. A range of extensive reviews that summarize the chemical synthesis, properties and applications of bimetallic nanoparticles are present in the literature (Xu *et al*., 2015; Zaleska‐Medynska *et al*., 2016; Loza *et al*., 2020). The emerging biotechnological application of microorganisms in synthesizing bimetallic nanoparticles offers several advantages over more traditional chemical synthesis routes. Biosynthesis presents a relatively simple, inexpensive, scalable and environmentally benign process for nanoparticle production (Lloyd *et al*., 2011). Biomolecules can act as capping agents, limiting nanoparticle aggregation while preventing oxidation with the biomass acting a structural support (Narayanan and Sakthivel, 2010; Bennett *et al*., 2013). Microbial processes can also be controlled during scale‐up to tune nanoparticle properties and in some cases can convert metal waste streams to a valuable end‐product, underpinning a transition towards a circular economy (Byrne *et al*., 2011; Byrne *et al*., 2015; Kimber *et al*., 2019). The ability of microorganisms to produce highly active monometallic catalysts is well known (De Windt *et al*., 2005; Lloyd *et al*., 2011; Kimber *et al*., 2018). However, the impact of microbial processes on the synthesis, properties and catalytic activity of bimetallic nanoparticles is not well understood. Several studies have investigated the biosynthesis of Pd‐based bimetallic nanoparticles, including Pd/Ru, Pd/Pt and Pd/Au, that demonstrated high catalytic activity (Deplanche *et al*., 2012; Murray *et al*., 2018; Omajali *et al*., 2019). However, these studies involved a two‐step synthesis process using cells ‘pre‐seeded’ with a metal component. *Shewanella oneidensis*, a common environmental bacterium, is able to precipitate a wide range of metals including Pd, Ag and Au via enzymatic reduction processes. Harnessing the metal‐reducing ability of this bacterium offers a potential one‐step / one‐pot process for bimetallic nanoparticle synthesis. Previous work explored the potential for microbially synthetized bimetallic Pd/Au nanoparticles to catalyse Suzuki coupling reactions (Heugebaert *et al*., 2012), but the corresponding Pd/Ag nanoparticles have not been studied, even though silver salts have been shown to enhance Pd‐catalysed Suzuki coupling (Zou *et al*., 2001; Mudarra *et al*., 2019). In particular, to the best of our knowledge, no studies exist on the application of biosynthesized Pd/Ag nanoparticles as Suzuki catalysts. Here, we investigate and compare for the first time the biosynthesis of bimetallic metal nanoparticles by cells of *S. oneidensis* challenged with multiple metals in solution (Pd/Ag or Pd/Au). Lactate was supplied as the sole electron donor to promote enzymatic reduction of metals and minimize abiotic H_2_ induced reduction. Detailed characterization of the nanoparticles was performed using a wide range of analytical techniques including scanning transmission electron microscopy (STEM) equipped with a high‐angle annular dark‐field (HAADF) detector, energy dispersive X‐ray analysis (EDX), X‐ray absorption near‐edge spectroscopy (XANES) and extended X‐ray absorption fine‐structure spectroscopy (EXAFS). The catalytic activity of the biosynthesized bimetallic nanoparticles was demonstrated against monometallic biosynthesized Pd nanoparticles in the Suzuki coupling reaction with excellent activity reported, particularly for the novel bio‐Pd/Ag nanoparticles. The generation of commercially relevant products is highlighted.

## Results and discussion

### Size analysis of Pd/Ag and Pd/Au bioprecipitates

**Fig. 1:**
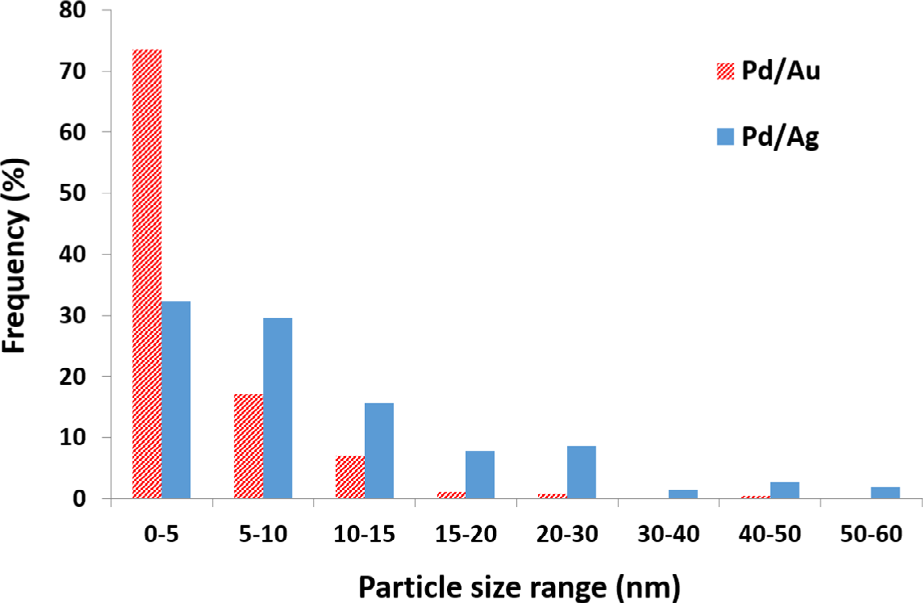
Histogram showing particle size distribution based on measurements of 257 randomly selected particles produced from Pd/Au and Pd/Au‐bearing solutions. The average particle size of bio‐Pd/Ag and bio‐Pd/Au was 11.2 ± 10.9 nm and 4.4 ± 4.5 nm respectively.

Pd/Au and Pd/Ag solutions were inoculated with cells of *S. oneidensis*. Lactate was supplied as the sole electron donor to promote enzymatic reduction of metals and minimize abiotic H_2_ induced reduction; H_2_ has been used in most previous microbial studies with Pd, although abiotic studies suggest that this leads to increased nanoparticle agglomeration and reduced activity (Yates *et al*., 2013). After 24 h incubation in the dark at 30 °C, ICP‐AES analysis confirmed that the majority of metals were removed from solution with 76% and 85% Pd(II) removed from the Pd/Ag and Pd/Au‐bearing solutions respectively (data not shown). Removal of Au and Ag was greater than 99% over the same time frame (data not shown). TEM imaging revealed clearly visible electron opaque precipitates after cells were challenged with either Pd/Ag or Pd/Au for 24 h (Fig. S1). Precipitates from the Pd/Ag system were clearly associated with the cell surface. When cells were challenged with Pd/Au, extracellular precipitates in close proximately to cells were also observed in addition to precipitates in direct contact with the cell. When no metals were added, no electron opaque regions were observed (Fig. S1). Particle size measurements suggest that the Pd/Au system resulted in a narrower size distribution range with 73.5% of measured particles less than 5 nm in size and 97.7% less than 15 nm in size (Fig. 1). The average particle size was determined to be 4.4 ± 4.5 nm. Previous work on the biosynthesis of Pd/Au nanoparticles by *S. oneidensis* using H_2_ as the electron donor resulted in a significantly wider size distribution of deposits with less than 50% of particles below 5 nm in size (De Corte *et al*., 2011) and a larger average particle size of 11.0 ± 13.7 nm. The smaller particle size in our study may be due to our omission of H_2_ as a potential electron donor which is known to abiotically reduce Pd(II) and result in larger, agglomerated particles (Yates *et al*., 2013). A wider particle size distribution was seen in precipitates from the Pd/Ag system with only 32 % of measured particles less than 5 nm, 77 % less than 15 nm, and 23 % between 15 nm and 60 nm. The average particle size of the Pd/Ag precipitates was 11.2 ± 10.9 nm. The biosynthesized nanoparticles from the Pd/Ag‐ and Pd/Au‐bearing solutions are herein referred to as bio‐Pd/Ag and bio‐Pd/Au respectively.

### Nanoparticle characterization using STEM‐HAADF and STEM‐EDX

**Fig. 2:**
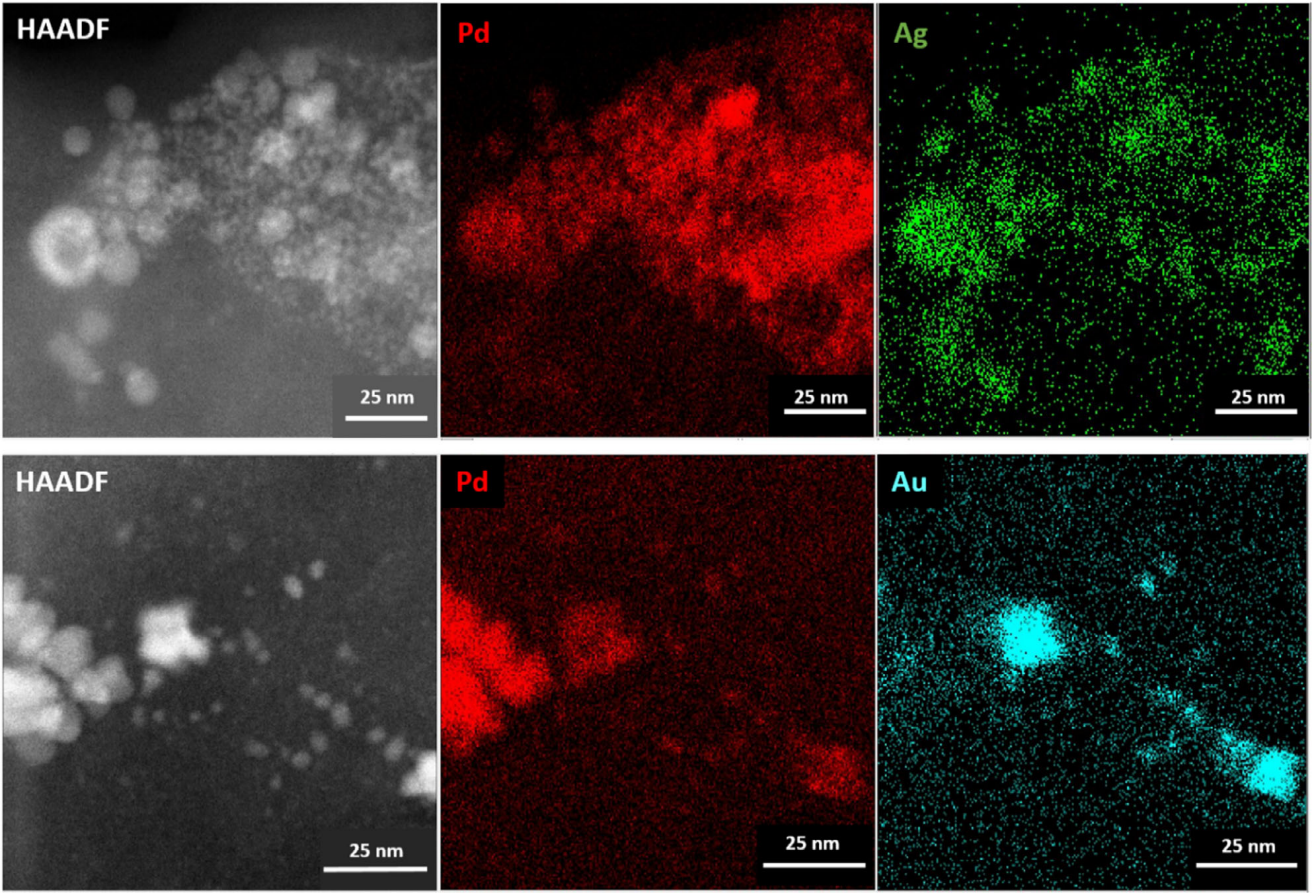
HAADF and STEM‐EDX images of biosynthesized nanoparticles from (top) Pd and Ag‐bearing solution and (bottom) Pd and Au‐bearing solution.

**Fig. 3:**
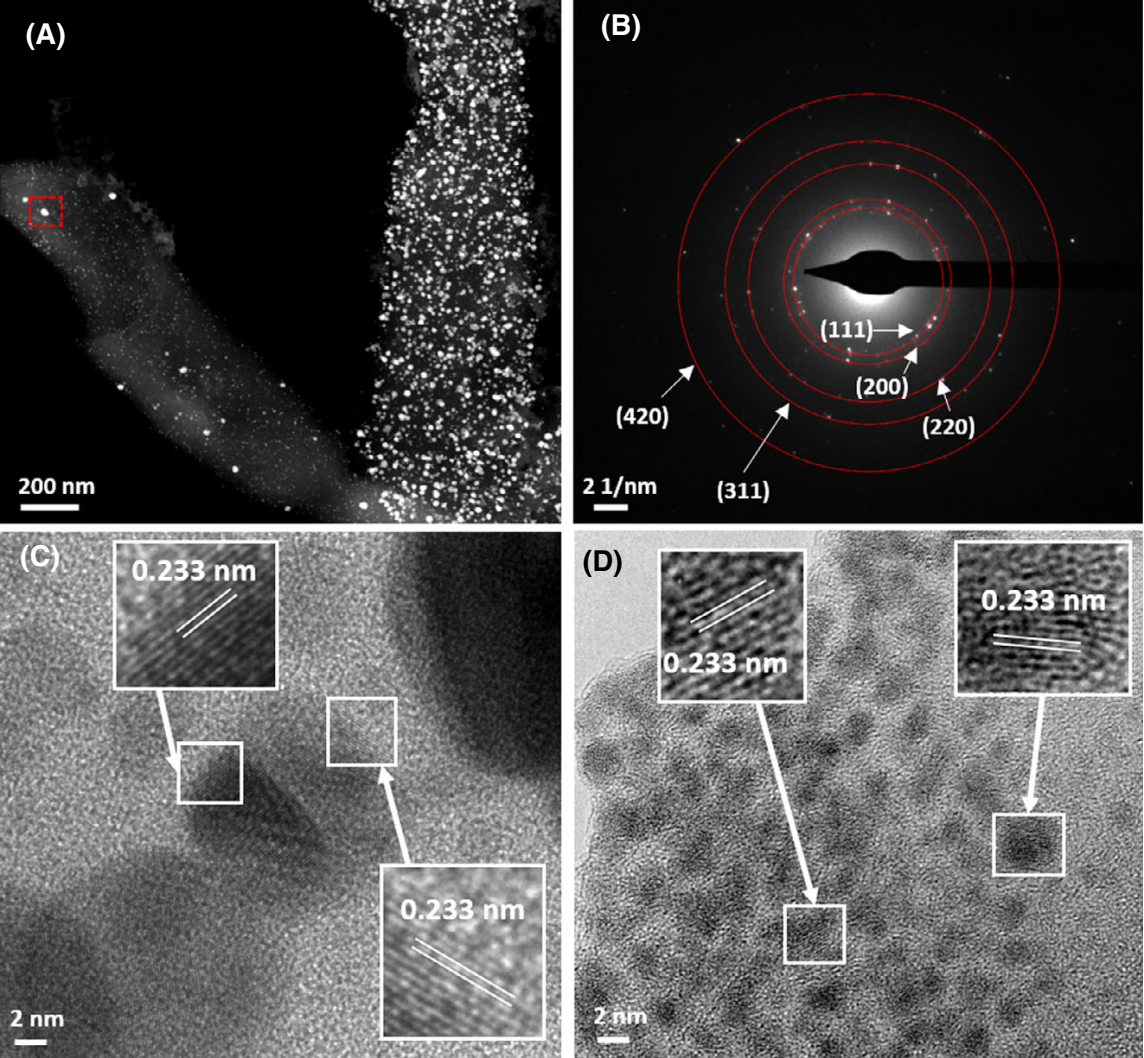
(A) STEM‐HAADF image of biosynthesized nanoparticles from Pd and Ag‐bearing solution and (B) corresponding polycrystalline select area diffraction pattern (from area highlighted in the red box) which is consistent with the FCC crystal structure of Ag(0). (C and D) Atomic resolution bright field images of small nanocrystals with corresponding lattice spacings consistent with the (111) facet of Pd(0).

STEM‐HAADF and EDX spot analysis suggests that the larger bio‐Pd/Ag nanoparticles were enriched in Ag and poor in Pd, while the smaller (< 10 nm) precipitates were enriched in Pd and poor in Ag (Fig. S2). A similar observation was made in the precipitates for bio‐Pd/Au, with larger (> 10 nm) particles Au rich and Pd poor (Fig. S3). However, the smaller (< 10 nm) bio‐Pd/Au particles appeared to show greater metal mixing than was seen for bio‐Pd/Ag. These observations are supported by STEM‐EDX mapping which revealed that both metals were present in the majority of bio‐Pd/Ag and bio‐Pd/Au but also highlights particle enrichment in individual metals (Fig. 2). The presence of Pd and Ag strongly correlated with particle size in the STEM‐EDX images, with larger particles clearly enriched in Ag and smaller particles dominated by Pd (Fig. S4). In addition, polycrystalline select area electron diffraction (SAED) patterns of these larger bio‐Pd/Ag nanoparticles are consistent with the FCC crystal structure of Ag(0) (Fig. 3A and B) while atomic resolution STEM‐HAADF images of the smaller particles reveal lattice spacings (0.233 nm) are consistent with the (111) facet of Pd(0) (Fig. 3C and D).

**Fig. 4:**
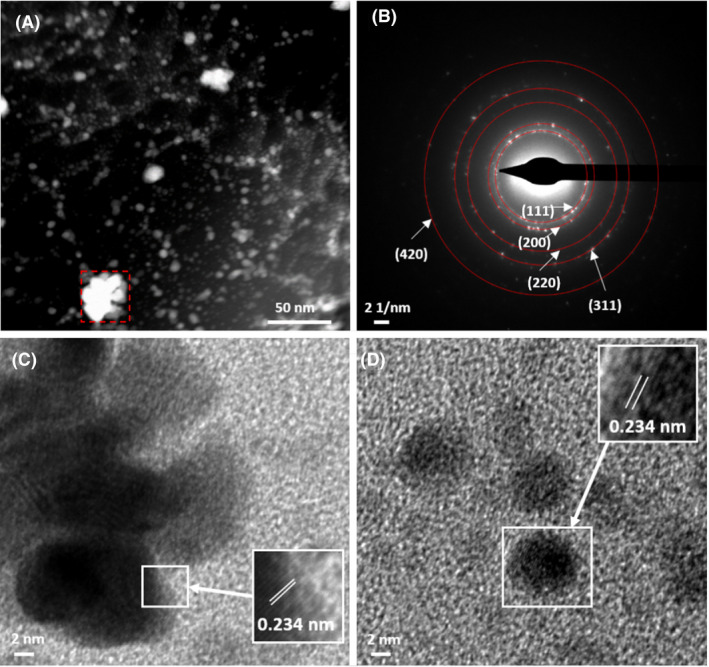
(A) STEM‐HAADF image of biosynthesized nanoparticles from Pd and Au‐bearing solution and (B) corresponding polycrystalline select area diffraction pattern (from area highlighted in the red box) which is consistent with the FCC crystal structure of Au(0). (C and D) Atomic resolution bright field images of small nanocrystals with corresponding lattice spacings consistent with the (111) facet of Pd(0).

For bio‐Pd/Au, STEM‐EDX mapping supports greater mixing of metals in the particles than was observed for bio‐Pd/Ag (Fig. S5). However, SAED patterns for the larger particles are consistent with the FCC crystal structure of Au(0) (Fig. 4A and B) with lattice spacings (0.234 nm) of the smaller particles again consistent with the (111) facet of Pd(0) (Fig. 4C and D).

**Fig. 5:**
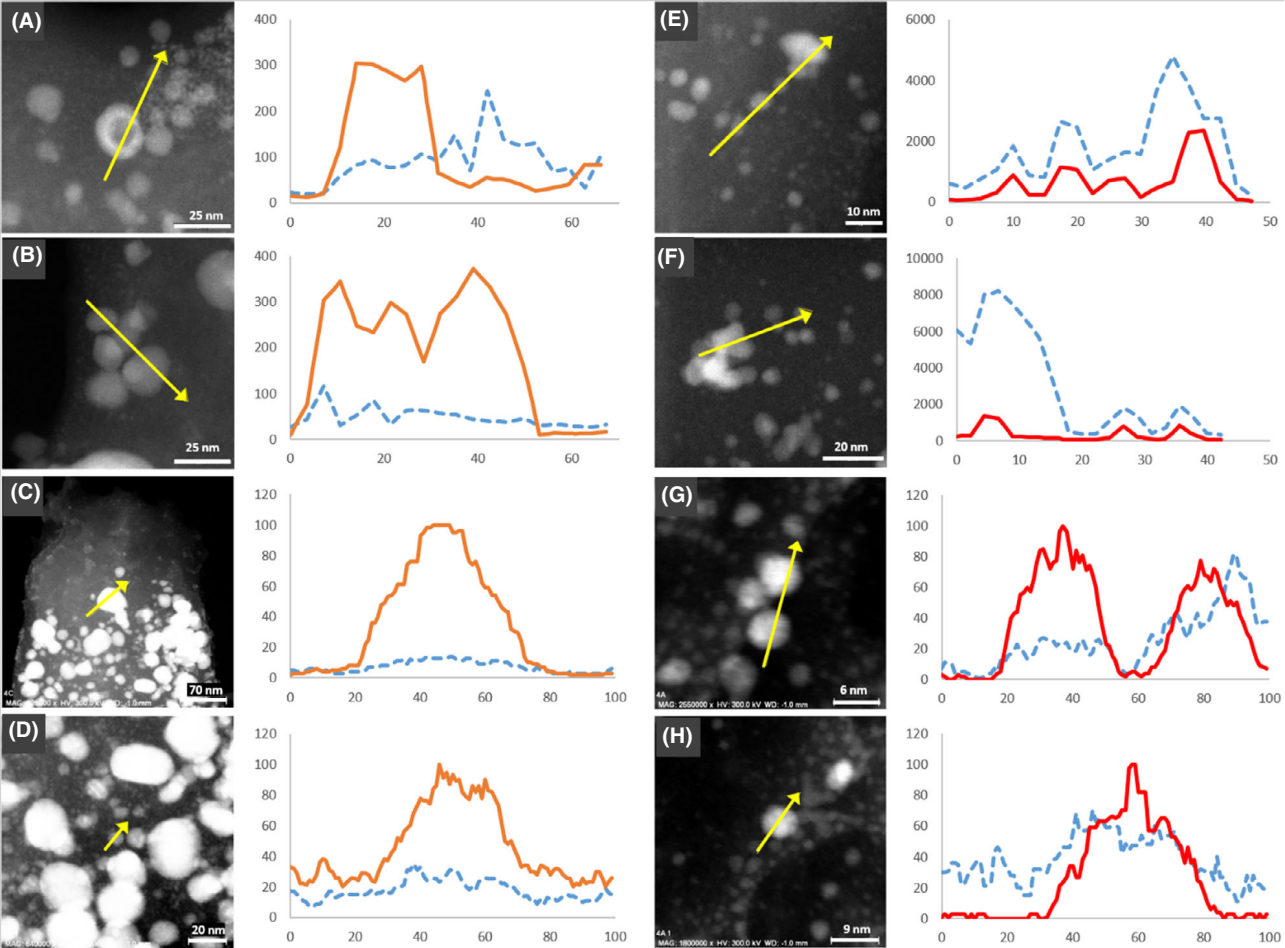
STEM‐HAADF image of biosynthesized nanoparticles from (A–D) Pd/Ag‐bearing solution and (E–H) Pd/Au‐bearing solution. Corresponding EDX line scans are shown to the right of the image. Pd is shown as the blue dashed line, Ag as the orange solid line and Au as the red solid line. The *y*‐axis represents counts, and values on the *x*‐axis are arbitrary units of distance.

Energy dispersive X‐ray line scans were performed on individual nanoparticles of both bio‐Pd/Ag and bio‐Pd/Au to further identify the composition and structure of the bioprecipitates. Although both metals were confirmed to be present in the bio‐Pd/Ag particles, a clear enrichment in Ag was again observed in larger particles with smaller nanoparticles (< 5 nm) showing enrichment in Pd (Fig. 5A–D). The largest particles (> 50 nm) appeared to show the highest enrichment of Ag relative to Pd (Fig. 5C) whereas particles in the 10–20 nm range, although still enriched in Ag, showed more mixing between metals (Fig. 5B and D). Interestingly, the formation of hollow Pd/Ag spheres were observed in STEM‐HAADF images (Fig. S6). An EDX transect through one of these hollow particles confirmed the particle is Ag rich and Pd poor, with the core depleted in both Pd and Ag relative to the surrounding shell (Fig. 5A).

EDX transects through bio‐Pd/Au again supported a much stronger correlation between Pd and Au than was observed for Pd and Ag (Fig. 5E). Interestingly, these transects also showed variations in the Pd and Au distributions which could indicate non‐uniform core–shell structure in a minority of particles (Fig. 5G–H). Previous authors have reported biosynthesis of Au@Pd core–shell nanoparticles using *Escherichia coli* (Deplanche *et al*., 2012); however, the *E. coli* cells were pre‐coated in Pd prior to Au(III) addition in contrast with the simultaneous reduction of both metals here.

### Nanoparticle characterization using bulk XAS: XANES analysis

**Fig. 6:**
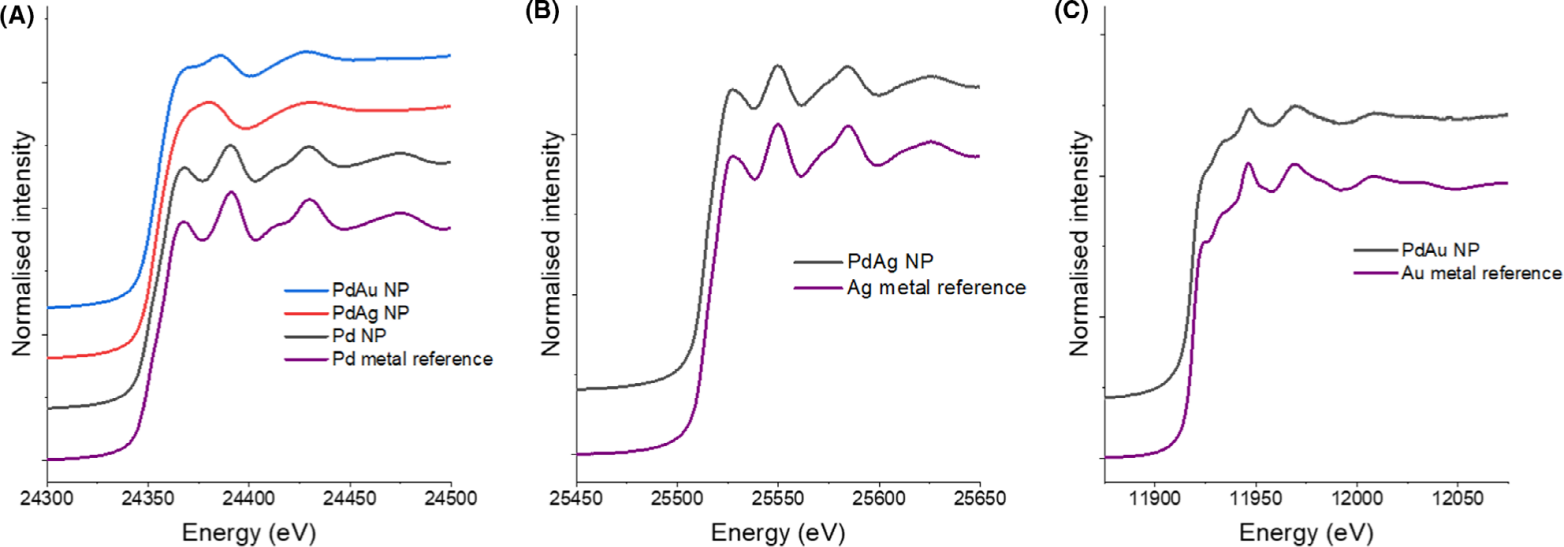
Offset XANES spectra showing the (A) Pd K‐edge of a Pdmetal reference foil and the Pd/Au, Pd/Ag and Pd only bioprecipitates; (B) Ag K‐edge of an Ag‐metal reference foil and the Pd/Ag bioprecipitates; and (C) Au L_3_ edge of a Au‐metal reference foil and the Pd/Au bioprecipitates.

The XANES region of the Pd K‐edge X‐ray absorption spectra of nanoparticles produced by cells challenged with Pd only, matched very closely with spectra from the metallic Pd foil reference, both with absorption edges located at 24 350 eV (Fig 6A). This is consistent with previous studies, clearly demonstrating biological reduction of Pd(II) to Pd(0), when Pd(II) is supplied as a lone metal (De Windt *et al*., 2005; Ng *et al*., 2013). Slightly smaller oscillations were observed in the post edge region in the XANES spectra of the bio‐Pd NPs compared to the Pd foil reference, consistent with their nanoparticulate nature. In contrast, the XANES collected from the Pd K‐edge of bio‐Pd/Ag and bio‐Pd/Au showed considerable differences to the Pd foil reference spectra, with absorption edges located at 24 353 eV (bio‐Pd/Ag) and 24 354 eV (bio‐Pd/Au). The broader white line peak in both the bio‐Pd/Ag and bio‐Pd/Au XANES spectra likely reflects a significant contribution of oxidized Pd(II), (Gomez‐Bolivar *et al*., 2019) suggesting incomplete reduction of Pd(II) or oxidation of Pd(0) in the sample. The bio‐Pd/Au spectrum showed a slightly closer resemblance to the Pd foil than the bio‐Pd/Ag spectrum suggesting greater reduction of Pd(II) to Pd(0) by the Pd/Au challenged cells compared to those challenged with Pd/Ag. However, in both cases it is likely from the significant differences in the XANES data that complete reduction of Pd(II) to Pd(0) is prevented when cells are challenged with an additional metal, either Ag(I) or Au(III). Alternatively, the presence of Ag(I) or Au(III) could promote abiotic oxidation of Pd(0), regenerating Pd(II) which was initially bioreduced.

The XANES spectra collected at the Ag K‐edge from the bio‐Pd/Ag nanoparticles showed a very strong match to that of an Ag foil reference, both with absorption edges located at 25 514 eV, confirming complete reduction of Ag(I) to Ag(0) by cells co‐supplied with Pd/Ag (Fig. 6b). The Au L_3_ edge XANES spectra of the Pd/Au nanoparticles showed a close resemblance to that of the Au foil reference, both with absorption edges at 11 919 eV and 2 main peaks located at 11 946 and 11 970 eV (Fig. 6C). However, differences in the white line shape and higher energy features were seen which could reflect the nanoparticulate size of bio‐Pd/Au.

### Pd K‐edge EXAFS analysis

**Fig. 7:**
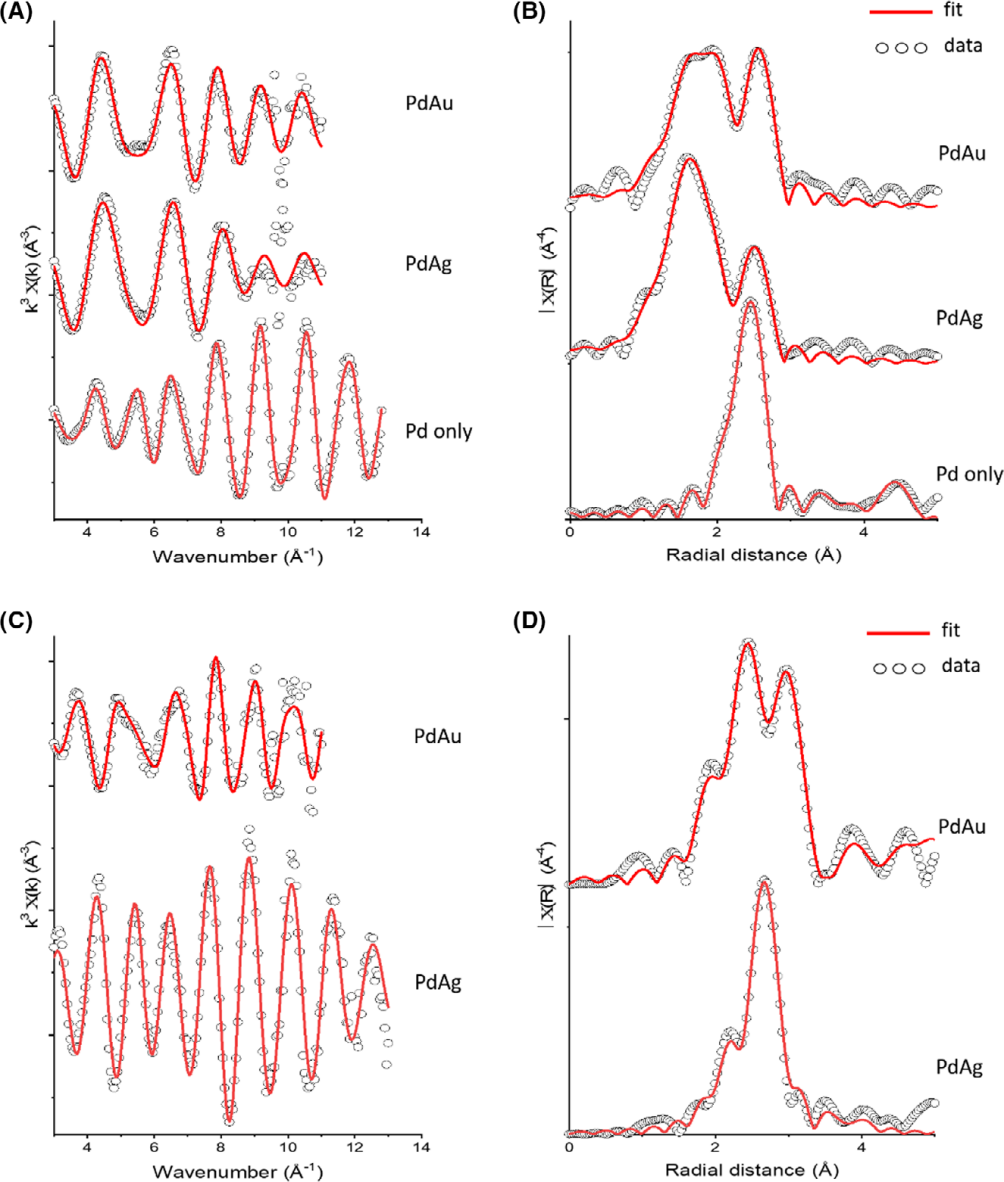
Pd K‐edge EXAFS (A) and Fourier transforms (B). From top to bottom: Pd/Au, Pd/Ag and Pd only bioprecipitates. Pd EXAFS has been reduced by a magnitude of 3 and FT EXAFS by a magnitude of 5 for direct comparison to the mixed metal systems. Au L3 edge and Ag K‐edge EXAFS (C) and Fourier Transforms (D). Pd/Au (top) and Pd/Ag (bottom). Ag FT EXAFS has been reduced by a magnitude of 3 for direct comparison to the Pd/Au systems.

Full EXAFS fitting parameters for all Pd K‐edge samples are provided in Table S1. Based on the XANES analysis, EXAFS data for the Pd only nanoparticles were fitted assuming a similar coordination environment as cubic Pd(0) (Fig. 7; Zemann, 1965). The best fit was obtained with three Pd–Pd shells with 10.5 Pd atoms coordinated at an interatomic distance of 2.74 Å, 6 Pd atoms at 3.88 Å, and 12 Pd atoms at 4.76 Å. The interatomic distance of the Pd–Pd shells is in excellent agreement with those of metallic Pd (2.75 Å, 3.89 Å, and 4.76 Å), further supporting the XANES analysis that complete bioreduction of Pd(II) to Pd(0) occurred in cell suspensions with monometallic Pd. A best fit was obtained with a reduced coordination number (CN) of 10.5 Pd atoms in the first shell at 2.74 Å, compared to the ideal structure of 12 Pd atoms in the first shell of metallic Pd, likely due to the reduced coordination of atoms near the nanoparticle surface (Marinković *et al*., 2016).

Fitting of the EXAFS spectra collected at the Pd K‐edge of bio‐Pd/Au and bio‐Pd/Ag included a Pd–Pd shell at 2.75 Å and 2.78 Å respectively. These Pd–Pd shells had much lower CNs than the corresponding Pd–Pd shell in the Pd only nanoparticles EXAFS fit, with only CNs of 3 in bio‐Pd/Au and 2 in the bio‐Pd/Ag. This suggests a significant non‐metallic contribution to the Pd EXAFS in both bio‐Pd/Au and bio‐Pd/Ag. The inclusion of O and S atoms at 2.0 and 2.3 Å, respectively, significantly improved the fitting, consistent with one or multiple non‐metallic Pd species being present. Full fitting parameters are provided in Table S1. It should be noted that the Pd–S shell could also be fitted as a Pd–P shell with similar fitting results, meaning that Pd–phosphine species may also be present and are difficult to distinguish from Pd–S (Table S2). EDX spot analysis and STEM‐EDX imaging support the presence of both P and S with Pd‐rich nanoparticles; however, a greater correlation between Pd and S was observed (Figs S2–S4). Previous authors have demonstrated that treatment of Pd materials with sulfur‐containing molecules, including thiols, can result in atomic S being incorporated into the subsurface region, yielding palladium–sulfide interphases such as Pd_4_S and Pd_3_S (Love *et al*., 2003; Zhang *et al*., 2015; Zhao *et al*., 2018). These interphases maintain high catalytic activity towards hydrogenation, as long as the catalyst does not become deactivated by full transformation to Pd–S (Zhang *et al*., 2015). Thiol groups of proteins from bacterial biomass have been shown to react with Pd, with deactivation of the Pd catalyst dependant on the concentration of the thiol‐containing protein (Søbjerg *et al*., 2011), offering a potential explanation for the formation of Pd–S coordination environments as indicated by our EXAFS data in this study. An interesting observation is that evidence of Pd–S coordination is only seen in bio‐Pd/Ag and bio‐Pd/Au and not in the Pd only control. The synthesis of Ag@Pd core–shell nanoparticles through galvanic exchange was found to substantially strengthen the chemisorption of Pd with reactants such as O_2_, promoting the transformation from Pd(0) to PdO (Li *et al*., 2018). Thus, the bimetallic structure of the bio‐Pd/Ag and bio‐Pd/Au here may have promoted similar transformation of Pd(0) to PdO or Pd–sulfide through enhanced reactivity with trace O_2_ or sulfur‐containing biomolecules, relative to the Pd only control.

Alloying of Pd with the larger Ag or Au atoms would be expected to increase the average bond length of the Pd‐metal shells. As the Pd–Pd bond length in bio‐Pd/Ag (2.75 Å) is in agreement with the Pd–Pd bond distance of Pd metal (2.75 Å), the EXAFS data suggest little alloying of Ag within the Pd structure. However, a slightly increased Pd–Pd bond distance of 2.78 Å is seen in the bio‐Pd/Au, in between that of Pd–Pd (2.75 Å) and Au–Au (2.88 Å) reference materials, and so may reflect limited alloying of Pd and Au. This is in agreement with previous authors who reported an increased Pd–Pd bond distance in Pd‐Au nanoparticles synthesized by *S. oneidensis* in the presence of H_2_ (De Corte *et al*., 2011) and is supported by EDX transects of bio‐Pd/Au in this study which show a good correlation between Pd and Au (Fig. 5).

### Ag K‐edge and Au L_3_ EXAFS analysis

EXAFS data collected at the Ag K‐edge from bio‐Pd/Ag were fitted with Ag–Ag bond lengths at 2.86 Å and 4.01 Å, slightly shorter than those of Ag metal at 2.89 Å and 4.09 Å. Substitution of smaller Pd atoms into the Ag‐rich nanoparticles may explain the reduced average Ag‐metal bond distances relative to pure Ag metal (Lahiri *et al*., 2005). The metallic structure revealed by the EXAFS spectra supports the XANES observations that complete reduction of Ag(I) occurred in the bimetallic Pd/Ag solution. For bio‐Pd/Au, the Au EXAFS spectra showed a greater deviation from that of the metal. A best fit was made with Au–Au shells at 2.85 Å, 3.64 Å, and 4.88 Å, shorter than the Au–Au distances in Au metal (2.88 Å, 4.08 Å, and 5.00 Å). These shorter Au–Au bond lengths are closer to the Pd–Pd distances in Pd metal (2.75 Å, 3.89 Å, and 4.76 Å) and so may reflect the substitution of Pd in the Au particle structure. However, the inclusion of Au–Pd shells did not statistically improve the fit. In addition, a statistically relevant Au‐S shell was fit with a low CN of 0.35 S atoms at a distance of 2.34 Å. Interestingly, the first Ag‐metal and Au‐metal shells were fitted with a significantly higher CN relative to the first Pd‐metal shell for both the bio‐Pd/Ag and bio‐Pd/Au samples. For bio‐Pd/Ag, the first Ag‐metal shell (Ag–Ag) was calculated with a CN of 10, 5 times higher than the CN of the corresponding first Pd‐metal shell (Pd–Pd). For bio‐Pd/Au, the CN of the first Au‐metal shell (Au–Au) was calculated to be 7, more than two times greater than the CN of the first Pd‐metal shell (Pd–Pd).

### Catalytic testing

Pd‐only bionanoparticles have previously been reported to show comparable activity to that of commercially available Pd catalysts (Bennett *et al*., 2013). Here, the catalytic potential of the biosynthesized bimetallic nanoparticles was tested by comparing their activity to that of bio‐Pd‐only nanoparticles in Suzuki–Miyaura cross‐coupling reactions, *i. e*.*,* Pd‐catalyzed C–C bond formation reactions between an aryl halide and an arylboronic acid. Suzuki–Miyaura coupling is a broadly applicable, scalable and cost‐effective step in the construction of the carbon skeleton of a wide range of pharmaceuticals, agrochemicals and fine chemical intermediates.

To determine the final metal loadings of the catalysts, the nanoparticle suspensions were washed several times with deionized water, and the pelleted samples were then subjected to an aqua regia digest. Bio‐Pd/Ag nanoparticles were found to comprise 44.8 wt% Pd and 55.2 wt% Ag. Bio‐Pd/Au nanoparticles contained 57.9 wt% Pd and 42.1 wt% Au (Table 1). A small amount of Au (1.2 wt%) was detected from the Pd‐only control digest, possibly from contamination during sampling. ICP‐AES measurements of the Pd‐only solution before and after bioreduction confirmed no Au was present in solution.

**Table 1:** Metal content of the biosynthesized nanoparticles in the final catalyst suspensions. The mass of catalyst used in each reaction test was normalized to 10 µg of Pd by adjusting the volume of the catalyst suspensions accordingly.

Biomaterial	Pd mass (mg/ml)	Metal loading (wt%)		
		Pd	Ag	Au
Pd only	0.835	98.8	N.D.	1.2
Pd/Au	0.761	57.9	N.D.	42.1
Pd/Ag	0.965	44.8	55.2	N.D.

The mass of Pd used in each reaction was normalized to 10 µg by adjusting the volume of the catalyst suspensions accordingly, based on metal loading data from the aqua regia digests. The coupling of *p*‐bromoacetophenone **2a** with phenylboronic acid **1a** was chosen as a model reaction for the optimization (Table 2). The reaction was performed in a sealed glass vial, in a mixed solvent (iPrOH/H_2_O) at 75 °C, under magnetic stirring. Conversions using the biosynthesized Pd‐only catalysts were < 5%, 11%, and 85% at 2, 5, and 24 h respectively. Both biosynthesized bimetallic nanoparticles significantly outperformed the Pd‐only control. Bio‐Pd/Au displayed a similarly low conversion of < 5% at 2 h but significantly greater conversions of 70% and > 99% at 5 and 24 hours respectively. Bio‐Pd/Ag performed even better, with 97% conversion by 2 h and > 99% conversion at 5 h. These data clearly show that Ag and Au significantly enhance the catalytic activity of Pd towards cross‐coupling reactions. Reactions performed using Au or Ag‐only nanoparticles synthesized by *S. oneidensis* resulted in no detectable conversions (Table 2), confirming that Au and Ag do not catalyse the reaction directly but rather act in synergy with Pd. Previous work has demonstrated the increased performance of bio‐Pd/Au nanoparticles, synthesized under a H_2_ atmosphere, relative to bio‐Pd (Heugebaert *et al*., 2012). Here, we show for the first time that bio‐Pd/Ag significantly outperforms both bio‐Pd and bio‐Pd/Au in the Suzuki–Miyaura cross‐coupling.

**Table 2:** Time‐course of the Suzuki–Miyaura cross‐coupling with biosynthesized nanoparticles. The volume of each catalyst suspension used in the reaction was adjusted to normalize Pd loading to 10 µg for 0.25 mmol **2a** in 5 ml (i.e. 0.04 %mol Pd loading). Conversions were determined by GC‐MS.

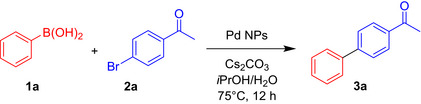			
Biosynthesized nanoparticles	Conv. (%)		
	2 h	5 h	24 h
Bio‐Pd only	< 5	11	85
Bio‐Pd/Ag	97	> 99	> 99
Bio‐Pd/Au	< 5	70	> 99
Bio‐Ag only	< 5	< 5	< 5
Bio‐Au only	< 5	< 5	< 5

**Scheme 1:**
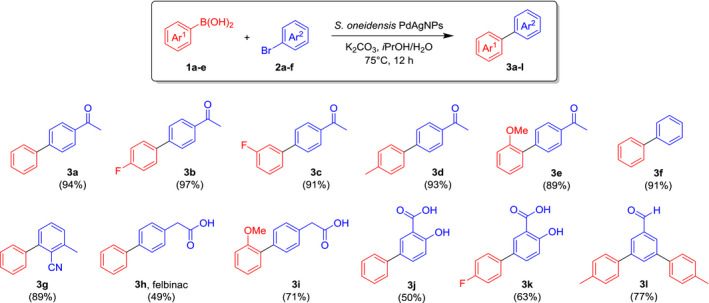
Preparative applications of the Suzuki–Miyaura cross‐coupling with biosynthesized PdAg‐NPs. Isolated yields are reported in brackets.

As bio‐Pd/Ag resulted in the greatest yield, significantly higher than that from the bio‐Pd‐only nanoparticles, we next investigated their activity towards the Suzuki–Miyaura cross‐coupling of a much broader range of arylboronic acids **1a–e** and aryl bromides **2a–f** on a preparative scale (Scheme 1). As expected, the reaction was found to be very versatile, tolerating different substituents (carbonyls, ethers, phenols, carboxylic acids, etc.). The products were obtained in good to excellent isolated yields (reported in Scheme 1), in some cases simply by recrystallization from the reaction solvent, otherwise by column chromatography. Even a double‐coupling reaction could be achieved simultaneously under the same conditions (with the substrate 3,5‐dibromobenzaldehyde, yielding the triaryl derivative **3l**). Conversions obtained for these reactions are reported in the Supporting Information (Table S4). Examples such as **3h** (the topical anti‐inflammatory felbinac) and **3j‐k** (analogues of the non‐steroidal painkiller diflunisal) are just representative of the outstanding practical importance of this type of transformation in the pharmaceutical, agrochemical and fine chemical industry. The development of more efficient, sustainable and environmentally friendly catalysts for Pd‐catalyzed couplings is a promising research area for the optimization and development of many chemical manufacturing processes.

## Conclusions

We demonstrated the biosynthesis of bimetallic Pd/Ag and Pd/Au nanoparticles using cells of *S. oneidensis* supplied with lactate as the sole electron donor. Bio‐Pd/Au nanoparticles showed a narrower size distribution than those of bio‐Pd/Ag. In addition, bio‐Pd/Au synthesized here using lactate, displayed a narrow size distribution and smaller average particle size than those synthesized under a H_2_ atmosphere in a previous study (De Corte *et al*., 2011), highlighting the importance of electron donor selection in controlling nanoparticle size. EDX analysis revealed the presence of both metals in nanoparticle deposits but suggested larger particles were enriched in Ag or Au with smaller particles enriched in Pd. TEM imaging and EDX transects suggested possible core–shell structures in bio‐Pd/Au and hollow Ag‐rich nanoparticles were observed in bio‐Pd/Ag. XAS analysis confirmed complete reduction of Pd(II) in Pd‐only controls, in agreement with previous studies. However, a significant contribution from Pd(II) was suggested from the Pd XANES spectra of the bio‐Pd/Ag and bio‐Pd/Au nanoparticles. EXAFS analysis confirmed a metallic Pd structure for the bio‐Pd‐only control but suggested multiple Pd species were present in the bimetallic nanoparticles, as inferred from Pd–Pd, Pd–O and Pd–S shells. The Pd–Pd and Pd–Ag bond distances in bio‐Pd/Ag suggested limited to no alloying of metals; however, bond distances of Pd–Pd and Au–Au in bio‐Pd/Au may suggest greater alloying of these two metals. Both bio‐Pd/Ag and bio‐Pd/Au demonstrated greatly enhanced catalytic activity towards Suzuki–Miyaura cross‐coupling than bio‐Pd only, with bio‐Pd/Ag significantly outperforming both. Bio‐Pd/Ag was shown to be versatile, tolerating a range of substituents and generating products with a good to excellent yields, including commercially relevant compounds. This demonstrates the potential of microorganisms for the green synthesis of highly active bimetallic nanoparticles with enhanced catalytic properties. Additional work is required to elucidate the mechanism responsible for increased catalytic performance. The efficacy of this biotechnological approach to synthesize NPs using other metal combinations, potentially from mixed metal waste sources, is also an avenue for further investigation.

## Experimental procedures

### Bimetallic nanoparticle synthesis

The experimental medium comprised of 30 ml solutions of MOPS buffer (50 mM) containing sodium lactate (30 mM) as the sole electron donor. The solutions were adjusted to pH 7.1, sealed with thick butyl rubber stoppers and purged with an 80 : 20 gas mix of N_2_:CO_2_ for 30 min to remove O_2_ and H_2_ and then autoclaved. Stock solution of Pd(II), Ag(I), and Au(III) was prepared using Na_2_PdCl_4_, AgNO_3_ and AuCl_3_ respectively. The metal salts were added to pre‐purged deionized water and then filter sterilized into pre‐autoclaved sealed serum bottles and purged again with N_2_:CO_2_ to remove any residual O_2_ and H_2_. Cells of *S. oneidensis* MR‐1 were grown as described previously (Kimber *et al*., 2018). Washed suspensions of late‐log harvested cells were added aseptically to the experimental medium to achieve a cell optical density (OD_600nm_) of 0.2. Metals stocks were then added to the medium to achieve the desired metal mix (Pd only, Pd/Ag, and Pd/Au) with equimolar concentrations of 200 µM. The bimetallic solutions (Pd/Ag and Pd/Au) were prepared via simultaneous addition of each metal. Following inoculation of metals, the solutions were incubated in the dark at 30 °C. After 24 h, metal solution concentrations in the bimetallic systems were determined using ICP‐AES.

### TEM and STEM imaging

TEM and STEM analysis was performed on samples taken from cell suspensions of *S. oneidensis* challenged with the bimetallic solutions (Pd/Ag and Pd/Au) following 24 hours incubation. One mL of cell suspensions was taken and centrifuged at 14 900 rpm for 5 min. The resulting supernatant was discarded, and the pellet resuspended in 1 ml deionized water. The cell suspension (1.5 µl) was pipetted onto a copper TEM grid with a holey‐carbon or carbon‐coated formvar support film and allowed to air dry in an anaerobic chamber. Samples were kept anaerobic until they were transferred into the TEM chamber, during which they would have been very briefly exposed to the atmosphere. TEM imaging and EDX analysis were performed either using an FEI Tecnai F30 FEG Analytical TEM operated at 300 kV or a JEM 2800 operated at 200 kV (0.20 nm STEM resolution) with two JEOL SDD Energy Dispersive Spectrometers. Additional STEM and EDX analyses were performed using a FEI image Cs‐corrector configuration Titan G2 60–300 STEM microscope equipped with HAADF detector, accelerating voltage of 300 kV available at the ‘Centro de Instrumentacion Cientifica’, at the University of Granada. Lattice spacings and SAED patterns from the collected images were analysed using Gatan DigitalMicrograph or ImageJ software (Schneider and Rasband, 2012), and particle size measurements were made using ImageJ.

### XAS characterization

XAS characterization was performed on the biosynthesized nanoparticles from the Pd/Ag, Pd/Au, and Pd‐only challenged cells. All manipulations were performed inside an anaerobic chamber. After 24 h incubation, 1 ml aliquots of the suspensions was taken and centrifuged (14 900 rpm for 5 min). The supernatant was discarded, and the pellet resuspended in 1 ml anoxic deionized water. A total of 200 µl of the suspension was pipetted onto a plastic weighing boat and air‐dried overnight. The dried samples were mounted onto a layer of kapton tape which was in turn mounted onto a plastic sample holder. A further layer of kapton tape was applied over the samples to maintain anoxic conditions. EXAFS spectra were collected at room temperature at the Pd K‐edge, Ag K‐edge, and Au L_3_ edge on beamline B18 at the Diamond Light Source. A 36‐element solid‐state Ge detector with digital signal processing for fluorescence EXAFS, high energy resolution and high count rate was used to measure with the beam at 45° incidence with respect to the sample holder plane. All spectra were acquired in quick‐EXAFS mode, using the Pt‐coated branch of collimating and focusing mirrors, a Si(111) double‐crystal monochromator and a pair of harmonic rejection mirrors. XANES processing was carried out using Athena while EXAFS data were modelled using Artemis (Demeter; 0.9.26; Ravel and Newville, 2005). Spectra were calibrated using a reference foil of the respective element, and the first inflection point of the white line was set to 24 350, 25 514 and 11 919 eV for Pd, Ag and Au respectively. Fitting was calculated using multiple k‐weights (k1, k2, and k3), and the best fit was calculated in R space by minimization of the reduced Χ^2^. At no point did parameterization of the EXAFS model use more than two‐thirds of the total number of available independent points. A shell‐by‐shell fitting process was employed, and additional shells were tested for statistical relevance using an ‘*F*‐test’ (Downward *et al*., 2009).

### Suzuki–Miyaura cross‐coupling

To a 20 ml glass vial was added the aryl bromide (0.25 mmol), the boronic acid (0.30 mmol, 1.2 equiv.) and potassium carbonate (104 mg, 0.75 mmol, 3 equiv.) or caesium carbonate (245 mg, 0.75 mmol, 3 equiv.). The solvent (iPrOH/H_2_O 1 : 1, 5 ml) was then added, and the mixture was stirred briefly. An aliquot of bio‐Pd/Ag or bio‐Pd/Au suspension (50–200 μl, equivalent to 10–100 μg Pd, as described in the text) was added to the vial, along with a stir bar. The vial was transferred to a sand bath over a hotplate and heated at 75°C for 12 h with constant stirring in order to keep the nanoparticles suspended. After cooling, in some cases the product separated out partially in the form of white or colourless crystals. The whole mixture was diluted with water (10 ml) and extracted with EtOAc (3 × 10 ml). The organic phases were combined, washed with brine (20 ml) and dried over anhydrous MgSO_4_. Evaporation of the solvent under reduced pressure afforded the product. Where needed, the product was purified by recrystallyzation (EtOH/H_2_O) or column chromatography (*n*‐hexane/EtOAc). Detailed characterization data and copies of the NMR and HRMS spectra of the products are reported in the Supporting Information.

## Conflict of interest

There are no conflicts to declare.

## Supporting information


**Fig. S1.** TEM and STEM images of *Shewanella oneidensis* cells after being challenged with (a‐c) no metals; (d‐e) Pd/Ag; and (f‐h) Pd/Au.
**Fig. S2.** HAADF‐STEM image and EDX spectra of selected nanoparticles synthesised from Pd and Ag bearing‐solution.
**Fig. S3.** HAADF‐STEM image and EDX spectra of selected nanoparticles synthesised from Pd and Au bearing‐solution.
**Fig. S4.** HAADF and EDX STEM images of nanoparticles synthesised from Pd and Ag bearing‐solution.
**Fig. S5.** HAADF and EDX STEM images of nanoparticles synthesised from Pd and Au bearing‐solution.
**Fig. S6.** HAADF STEM images of nanoparticles synthesised from Pd and Ag bearing‐solution highlighting the formation of hollow nanoparticle spheres.
**Table S1.** EXAFS fitting parameters for Pd‐K edge EXAFS of the Pd, PdAu and PdAg bioprecipitates. Coordination numbers (N), U bond distances (R (Å)), shift in energy from calculated Fermi level (ΔE0), Debye−Waller factors (σ2), amplitude reduction factors (S0) and “goodness of fit” factor (R). Coordination numbers were fixed. Numbers in parentheses are the standard deviation on the last decimal place.
**Table S2.** EXAFS fitting parameters for Pd‐K edge EXAFS of the Pd, Pd Au and PdAg bioprecipitates including a P shell. Coordination numbers (N), U bond distances (R (Å)), shift in energy from calculated Fermi level (ΔE0), Debye−Waller factors (σ2), amplitude reduction factors (S0) and “goodness of fit” factor (R). Coordination numbers were fixed. Numbers in parentheses are the standard deviation on the last decimal place.
**Table S3.** EXAFS fitting parameters for Ag K and Au L3 edge EXAFS of the Pd Au and PdAg bioprecipitates. Coordination numbers (N), U bond distances (R (Å)), shift in energy from calculated Fermi level (ΔE0), Debye−Waller factors (σ2), amplitude reduction factors (S0) and “goodness of fit” factor (R). Coordination numbers were fixed. Numbers in parentheses are the standard deviation on the last decimal place.
**Table S4.** Conversions for the preparative‐scale Suzuki–Miyaura cross‐coupling reactions with biosynthesised PdAg‐NPs (as described in the text). Conversion values are calculated by GC‐MS analysis.
**Data S1.** Characterization data of the products and copies of the NMR and HRMS spectra.
