# Supplementary material for: Biotechnological synthesis of Pd/Ag and Pd/Au nanoparticles for enhanced Suzuki–Miyaura cross‐coupling activity
Source: Microb Biotechnol. 2021 Mar 15;14(6):2435–47. doi: 10.1111/1751-7915.13762 (PMC8601183; doi:10.1111/1751-7915.13762)

**Biotechnological synthesis of Pd/Ag and Pd/Au nanoparticles for enhanced Suzuki-Miyaura cross-coupling activity**

Richard L. Kimber^1a^, Fabio Parmeggiani^2b^, Thomas S. Neill^1c^, Mohamed L. Merroun^3^, Gregory Goodlet^4^, Nigel A. Powell^4^, Nicholas J. Turner^2^, Jon R. Lloyd^1^

^1^ Department of Earth and Environmental Sciences and Williamson Research Centre for Molecular Environmental Science, University of Manchester, Manchester, UK

^2^ Department of Chemistry, Manchester Institute of Biotechnology (MIB), University of Manchester, Manchester, UK

^3^ Department of Microbiology, Faculty of Sciences, University of Granada, Campus Fuentenueva, 18071 Granada, Spain

^4^ Johnson Matthey Technology Centre, Reading, RG4 9NH, UK

^a^ Department of Environmental Geosciences, University of Vienna, Althanstraße 14 (UZA II), 1090, Vienna, Austria

^b^ Department of Chemistry, Materials and Chemical Engineering “G. Natta”, Politecnico di Milano, Via Mancinelli 7, 20131, Milano, Italy.

^c^ Institute for Nuclear Waste Disposal, Karlsruhe Institute of Technology, 76021 Karlsruhe, Germany

**SUPPORTING INFORMATION**

**TABLE OF CONTENTS**

Supplementary figures pages 3-8

Supplementary tables pages 9-11

Analytical methods pages 12

Compound characterisation data pages 13-15

Copies of NMR and HRMS spectra pages 16-39

**Supplementary figures**


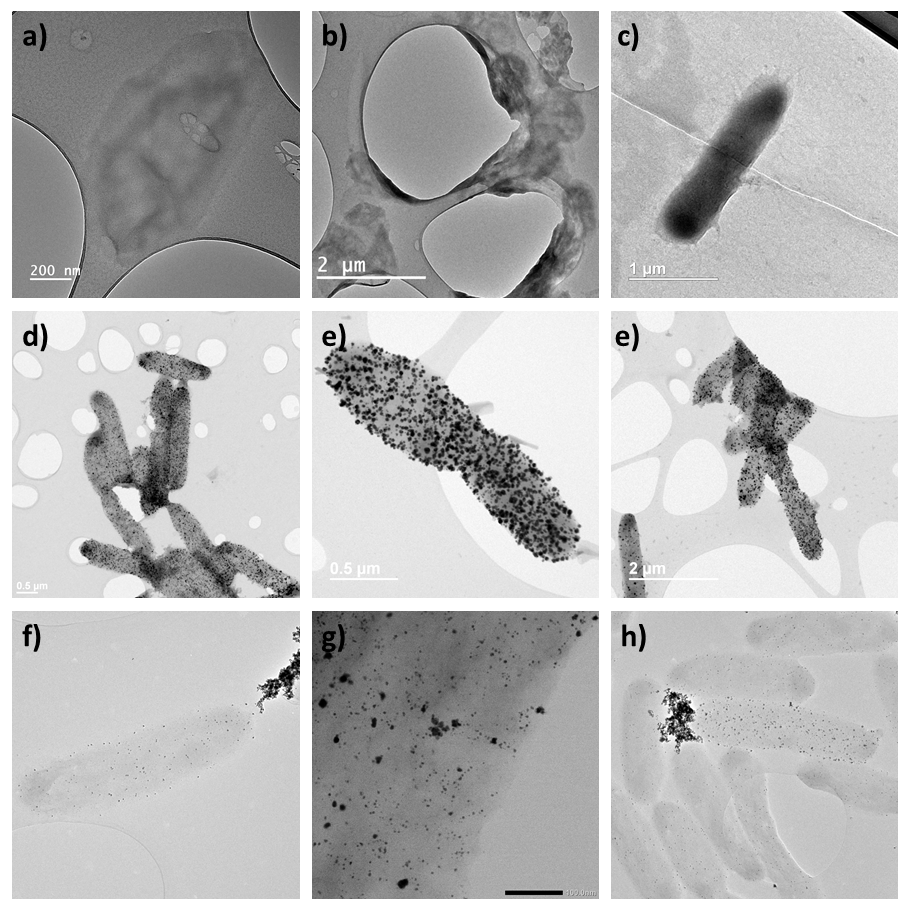


**Figure S1.** TEM and STEM images of *Shewanella oneidensis* cells after being challenged with **(a-c)** no metals; **(d-e)** Pd/Ag; and **(f-h)** Pd/Au.


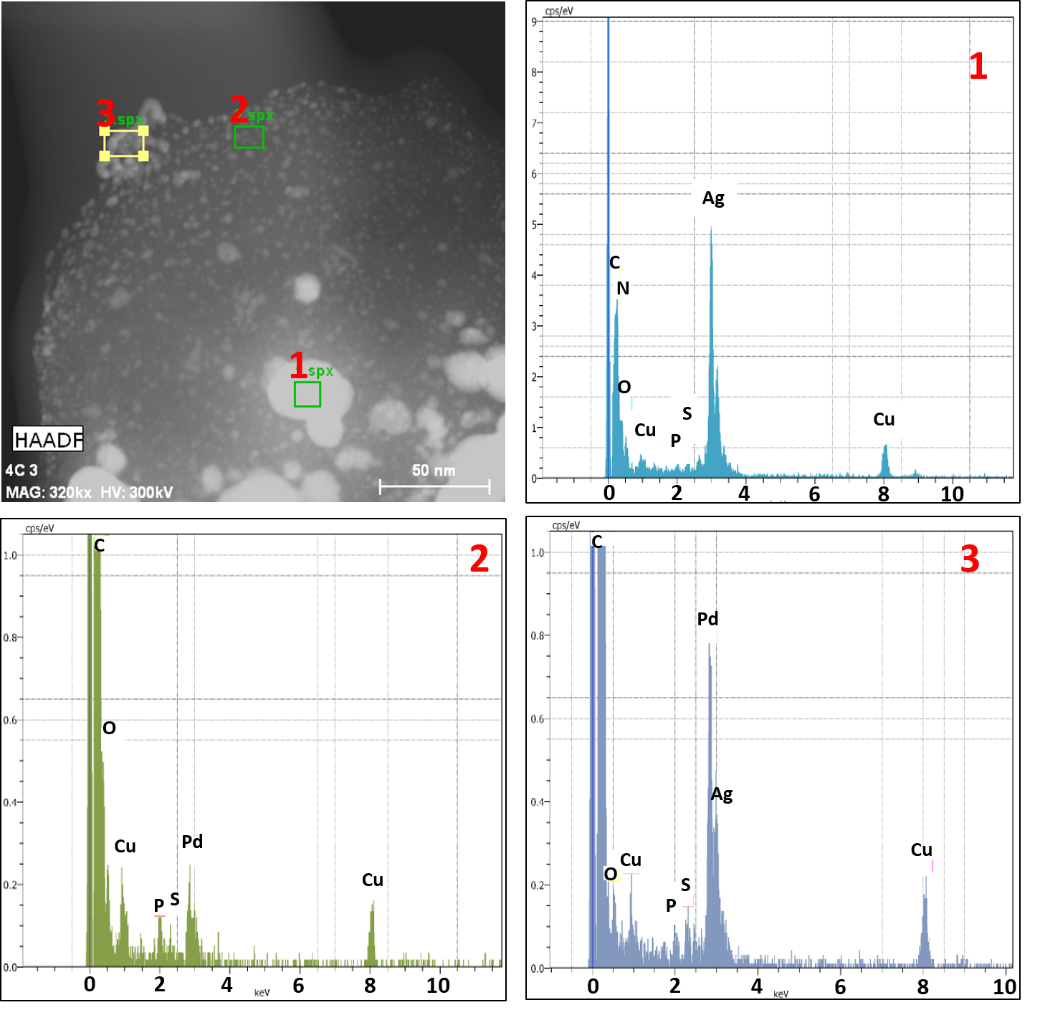


**Figure S2.** HAADF-STEM image and EDX spectra of selected nanoparticles synthesised from Pd and Ag bearing-solution.


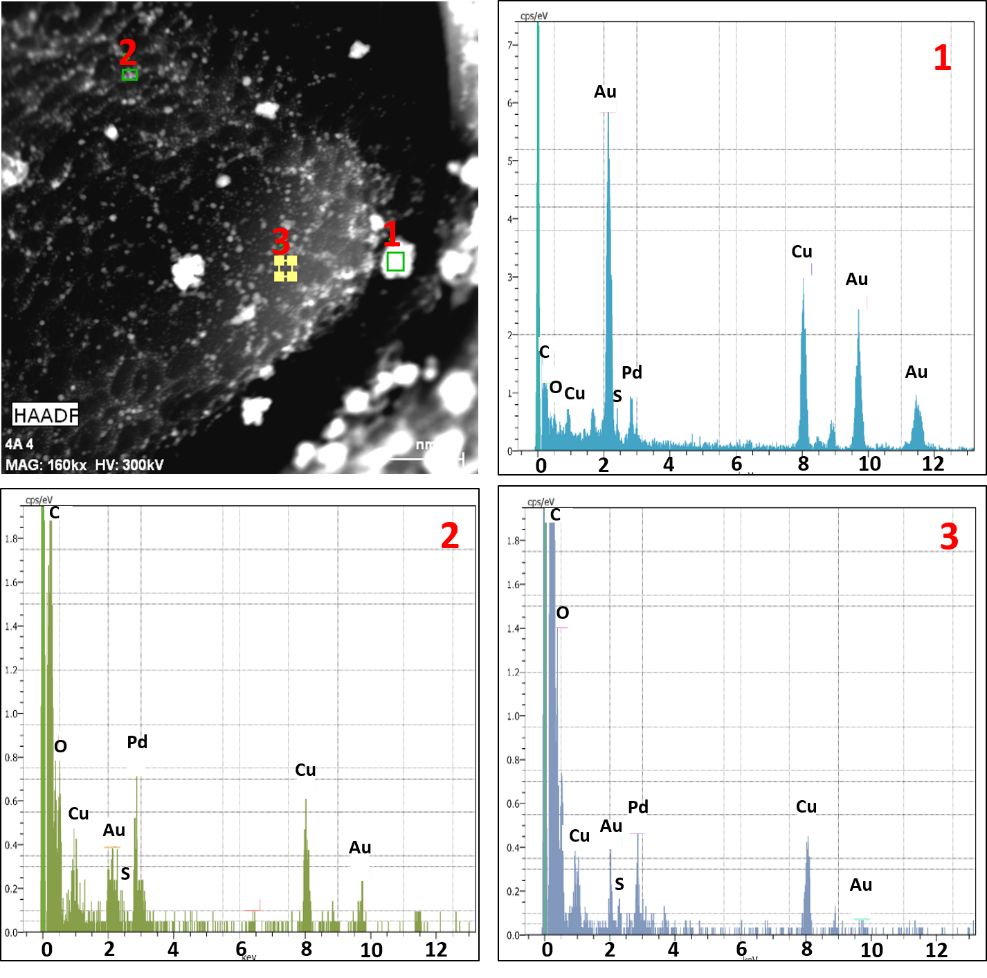


**Figure S3.** HAADF-STEM image and EDX spectra of selected nanoparticles synthesised from Pd and Au bearing-solution.


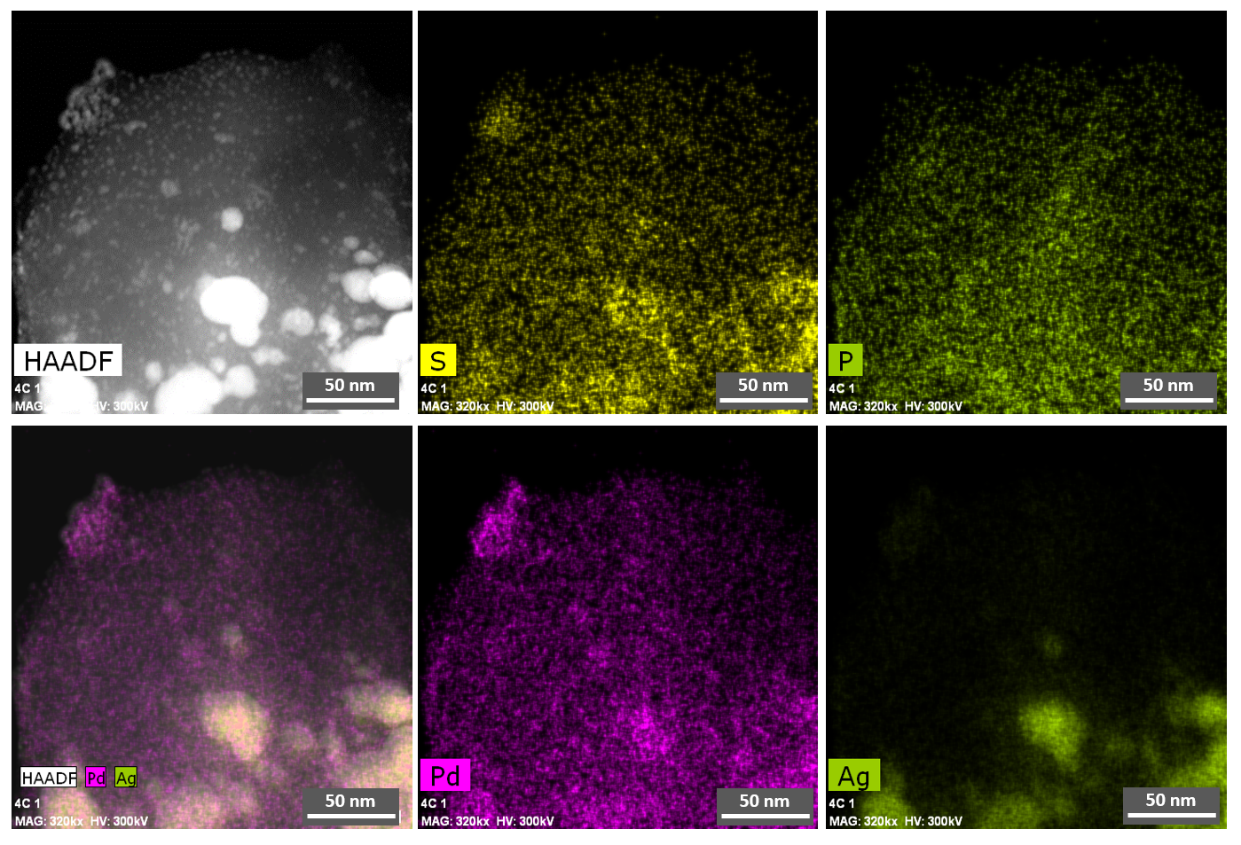


**Figure S4.** HAADF and EDX STEM images of nanoparticles synthesised from Pd and Ag bearing-solution.


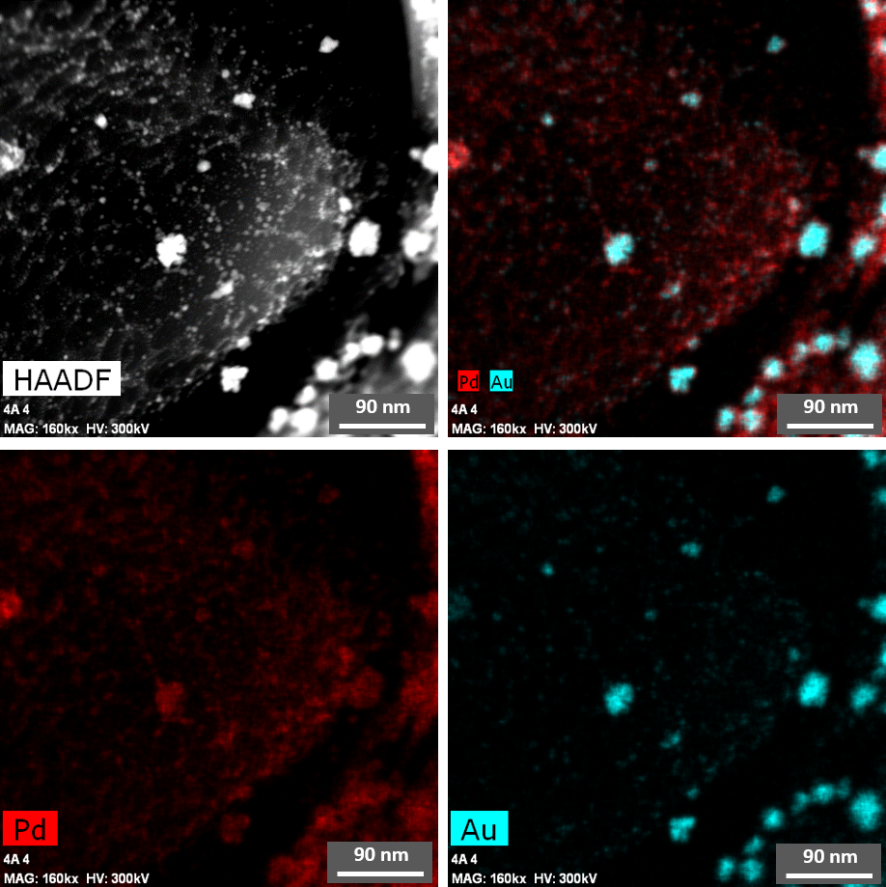


**Figure S5.** HAADF and EDX STEM images of nanoparticles synthesised from Pd and Au bearing-solution.


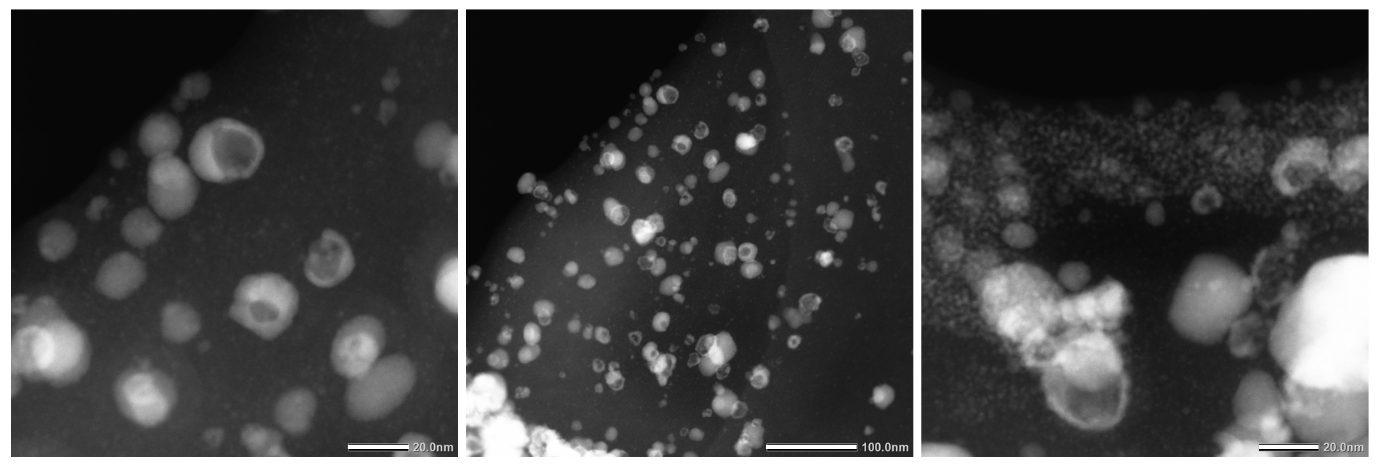


**Figure S6.** HAADF STEM images of nanoparticles synthesised from Pd and Ag bearing-solution highlighting the formation of hollow nanoparticle spheres.

**Supplementary tables**

**Table S1.** EXAFS fitting parameters for Pd-K edge EXAFS of the Pd, PdAu and PdAg bioprecipitates. Coordination numbers (N), U bond distances (R (Å)), shift in energy from calculated Fermi level (ΔE0), Debye−Waller factors (σ2), amplitude reduction factors (S0) and “goodness of fit” factor (R). Coordination numbers were fixed. Numbers in parentheses are the standard deviation on the last decimal place.

| **Sample** | **Shell** | **N** | **R (Å)** | **ΔE_0_ (eV)** | **σ^2^ (Å^2^)** | **S_0_** | **R factor** |
| --- | --- | --- | --- | --- | --- | --- | --- |
| **Biometallic Pd** | Pd.1 | 10.5 | 2.74(0) | -6.6(5) | 0.0068(2) | 0.75 | 0.013 |
|  | Pd.2 | 6 | 3.88(2) | -6.6(5) | 0.0114(20) |  |  |
|  | Pd.3 | 12 | 4.76(1) | -6.6(5) | 0.0091(13) |  |  |
|  | Pd ms | 48 | 4.12(5) | -6.6(5) | 0.0101(3) |  |  |
| **PdAu** | O.1 | 1.3 | 2.02(3) | 3.3(10) | 0.0134(50) | 0.70 | 0.012 |
|  | S.1 | 2.5 | 2.34(1) | 3.3(10) | 0.0090(17) |  |  |
|  | Pd.1 | 3 | 2.78(1) | 3.3(10) | 0.0123(9) |  |  |
| **PdAg** | O.1 | 2 | 2.00(3) | 0.4(1.7) | 0.0133(47) | 0.80 | 0.010 |
|  | S.1 | 2 | 2.29(2) | 0.4(1.7) | 0.0095(25) |  |  |
|  | Pd.1 | 2 | 2.75(2) | 0.4(1.7) | 0.0099(26) |  |  |

**Table S2.** EXAFS fitting parameters for Pd-K edge EXAFS of the Pd, Pd Au and PdAg bioprecipitates including a P shell. Coordination numbers (N), U bond distances (R (Å)), shift in energy from calculated Fermi level (ΔE0), Debye−Waller factors (σ2), amplitude reduction factors (S0) and “goodness of fit” factor (R). Coordination numbers were fixed. Numbers in parentheses are the standard deviation on the last decimal place.

| **Sample** | **Shell** | **N** | **R (Å)** | **ΔE_0_ (eV)** | **σ^2^ (Å^2^)** | **S_0_** | **R factor** |
| --- | --- | --- | --- | --- | --- | --- | --- |
| **PdAu** | O.1 | 1.3 | 2.02(3) | 3.7(10) | 0.0099(36) | 0.7 | 0.012 |
|  | P.1 | 2.5 | 2.37(1) | 3.7(10) | 0.0111(17) |  |  |
|  | Pd.1 | 3 | 2.78(1) | 3.7(10) | 0.0091(10) |  |  |
| **PdAg** | O.1 | 2 | 1.99(3) | 0.7(17) | 0.0106(33) | 0.8 | 0.010 |
|  | P.1 | 2 | 2.32(2) | 0.7(17) | 0.0087(24) |  |  |
|  | Pd.1 | 2 | 2.75(2) | 0.7(17) | 0.0100(26) |  |  |

**Table S3.** EXAFS fitting parameters for Ag K and Au L3 edge EXAFS of the Pd Au and PdAg bioprecipitates. Coordination numbers (N), U bond distances (R (Å)), shift in energy from calculated Fermi level (ΔE0), Debye−Waller factors (σ2), amplitude reduction factors (S0) and “goodness of fit” factor (R). Coordination numbers were fixed. Numbers in parentheses are the standard deviation on the last decimal place.

| **Sample** | **Shell** | **N** | **R (Å)** | **ΔE_0_ (eV)** | **σ^2^ (Å^2^)** | **S_0_** | **R factor** |
| --- | --- | --- | --- | --- | --- | --- | --- |
| **PdAu** | S.1 | 0.35 | 2.34(4) | 4.2(7) | 0.0099(53) | 0.9 | 0.020 |
|  | Au.1 | 7 | 2.85(1) | 4.2(7) | 0.0110(6) |  |  |
|  | Au.2 | 3 | 3.64(3) | 4.2(7) | 0.0144(41) |  |  |
|  | Au.3 | 6 | 4.88(4) | 4.2(7) | 0.0117(54) |  |  |
| **PdAg** | Ag.1 | 10 | 2.86(0) | 3.3(4) | 0.0111(5) | 0.88 | 0.014 |
|  | Ag.2 | 3 | 4.01(3) | 3.3(4) | 0.0130(40) |  |  |
|  |  |  |  |  |  |  |  |

**Table S4.** Conversions for the preparative-scale Suzuki-Miyaura cross-coupling reactions with biosynthesised PdAg-NPs (as described in the text). Conversion values are calculated by GC-MS analysis.

| **Product** | **Conversion [%]**  **10 μg Pd** | **Conversion [%]**  **100 μg Pd** |
| --- | --- | --- |
| **3a** | >99 | –^a^ |
| **3b** | >99 | –^a^ |
| **3c** | >99 | –^a^ |
| **3d** | >99 | –^a^ |
| **3e** | >99 | –^a^ |
| **3f** | >99 | –^a^ |
| **3g** | >99 | –^a^ |
| **3h** | 37 | 55 |
| **3i** | 81 | –^a^ |
| **3j** | 39 | 56 |
| **3k** | 44 | 68 |
| **3l** | 79 | 85 |

^a^ Not tested.

**Analytical methods**

**Analytical methods**

^1^H and ^13^C NMR spectra were recorded on a Bruker Avance 400 NMR spectrometer (400 MHz) at 298 K. Chemical shifts are reported as δ in parts per million (ppm) and are calibrated against residual solvent signal. A water suppression method was used to obtain the ^1^H NMR spectra of carboxylic acids in D_2_O (basified with a small amount of NaOH), with HDO as the residual solvent peak (4.79 ppm). NMR data are reported as follows: chemical shift (ppm), multiplicity (s = singlet, d = doublet, t = triplet, q = quartet, m = multiplet), coupling constants (Hz) and proton integration.

HRMS analyses were performed using an Agilent 1200 series LC system, coupled to an Agilent 6520 QTOF mass spectrometer, ESI positive mode. The sample (2 µL) was flow-injected into 0.3 mL min^–1^ MeCN/H_2_O 1:1 + formic acid 0.1% v/v. The data was analyzed using Agilent MassHunter software.

GC-MS analyses were performed on an Agilent 7980B GC 5977B MSD system equipped with a HP-5-MS column (30 m × 0.25 mm × 0.25 µm, Agilent), according to the following temperature program: 60°C (1 min), 6°C/min, 150°C (1 min), 12°C/min, 280°C (5 min).

**Compound characterisation data**

**1-([1,1'-biphenyl]-4-yl)ethan-1-one (3a)**

From 4’-bromoacetophenone and phenylboronic acid.

White crystals, 46 mg (94% isolated yield).

**^1^H NMR** (400 MHz, CDCl_3_): δ 8.04 (d, *J*=8.1, 2H), 7.69 (d, *J*=8.1, 2H), 7.66-7.61 (m, 2H), 7.51-7.45 (m, 2H), 7.43-7.38 (m, 3H), 2.65 (s, 3H).

**^13^C NMR** (101 MHz, CDCl_3_): δ 197.9, 145.9, 140.0, 136.0, 129.1, 129.0, 128.3, 127.4, 127.3, 26.8.

**GCMS** (EI): 152 (61), 181 (100), 196 (52) [M]^+^; *t_R_* = 19.51 min.

**HRMS** (ESI): *m/z* for C_14_H_13_O^+^ [M+H]^+^ calcd. 197.0961, found 197.0965.

**1-(4'-fluoro-[1,1'-biphenyl]-4-yl)ethan-1-one (3b)**

From 4’-bromoacetophenone and *p*-fluorophenylboronic acid.

White crystals, 52 mg (97% isolated yield).

**^1^H NMR** (400 MHz, CDCl_3_): δ 8.02 (d, *J*=8.2, 2H), 7.63 (d, *J*=8.2, 2H), 7.61-7.55 (m, 2H), 7.19-7.12 (m, 2H), 2.64 (s, 3H).

**^13^C NMR** (101 MHz, CDCl_3_): δ 197.8, 163.1 (d, *J*=248.5), 144.8, 136.1 (d, *J*= 3.0), 136.0, 129.1, 129.0, 127.2, 116.0 (d, *J*=21.5), 26.8.

**GCMS** (EI): 170 (71), 199 (100), 214 (55) [M]^+^; *t_R_* = 19.58 min.

**HRMS** (ESI): *m/z* for C_14_H_12_FO^+^ [M+H]^+^ calcd. 215.0867, found 215.0872.

**1-(3'-fluoro-[1,1'-biphenyl]-4-yl)ethan-1-one (3c)**

From 4’-bromoacetophenone and *m*-fluorophenylboronic acid.

White crystals, 49 mg (xx91% isolated yield).

**^1^H NMR** (400 MHz, CDCl_3_): δ 8.04 (d, *J*=8.4, 2H), 7.66 (d, *J*=8.4, 2H), 7.47-7.38 (m, 2H), 7.35-7.29 (m, 1H), 7.13-7.05 (m, 1H), 2.64 (s, 3H).

**^13^C NMR** (101 MHz, CDCl_3_): δ 197.7, 163.3 (d, *J*=246.6), 144.5 (d, *J*=2.1), 142.2 (d, *J*=7.7), 136.4, 130.6 (d, *J*=8.4), 129.1, 127.3, 123.0 (d, *J*=2.9), 115.1 (d, *J*=21.2), 114.3 (d, *J*=22.4), 26.8.

**GCMS** (EI): 170 (53), 199 (100), 214 (48) [M]^+^; *t_R_* = 19.48 min.

**HRMS** (ESI): *m/z* for C_14_H_12_FO^+^ [M+H]^+^ calcd. 215.0867, found 215.0870.

**1-(4'-methyl-[1,1'-biphenyl]-4-yl)ethan-1-one (3d)**

From 4’-bromoacetophenone and *p*-tolylboronic acid.

White crystals, 49 mg (93% isolated yield).

**^1^H NMR** (400 MHz, CDCl_3_): δ 8.02 (d, *J*=8.4, 2H), 7.67 (d, *J*=8.4, 2H), 7.54 (d, *J*=8.1, 2H), 7.28 (d, *J*=8.1, 2H), 2.63 (s, 3H), 2.42 (s, 3H).

**^13^C NMR** (101 MHz, CDCl_3_): δ 197.9, 145.8, 138.4, 137.1, 135.7, 129.8, 129.0, 127.22, 127.1, 26.8, 21.30.

**GCMS** (EI): 152 (34), 165 (26), 195 (100), 210 (53) [M]^+^; *t_R_* = 21.03 min.

**HRMS** (ESI): *m/z* for C_15_H_15_O^+^ [M+H]^+^ calcd. 211.1117, found 211.1122.

**1-(2'-methoxy-[1,1'-biphenyl]-4-yl)ethan-1-one (3e)**

From 4’-bromoacetophenone and *o*-methoxyphenylboronic acid.

Pale yellow crystals, 51 mg (89% isolated yield).

**^1^H NMR** (400 MHz, CDCl_3_): δ 8.04 (d, *J*=8.5, 2H), 7.64 (d, *J*=8.5, 2H), 7.41-7.32 (m, 2H), 7.09-7.03 (m, 1H), 7.03-6.99 (m, 1H), 3.83 (s, 3H), 2.64 (s, 3H).

**^13^C NMR** (101 MHz, CDCl_3_): δ 198.0, 256.6, 143.7, 135.6, 130.8, 129.8, 129.6, 129.5, 128.2, 121.1, 111.5, 55.7, 26.8.

**GCMS** (EI): 139 (21), 168 (47), 211 (100), 226 (60) [M]^+^; *t_R_* = 21.58 min.

**HRMS** (ESI): *m/z* for C_15_H_15_O_2_^+^ [M+H]^+^ calcd. 227.1067, found 227.1071.

**1,1'-biphenyl (3f)**

From bromobenzene and phenylboronic acid.

White crystals, 35 mg (91% isolated yield).

**^1^H NMR** (400 MHz, CDCl_3_): δ 7.66-7.59 (m, 4H), 7.50-7.42 (m, 4H), 7.40-7.33 (m, 2H).

**^13^C NMR** (101 MHz, CDCl_3_): δ 141.4, 128.9, 127.4, 127.3.

**GCMS** (EI): 76 (9), 154 (100) [M]^+^; *t_R_* = 11.57 min.

**3-methyl-[1,1'-biphenyl]-2-carbonitrile (3g)**

From 2-bromo-6-methylbenzonitrile and phenylboronic acid.

Pale yellow solid, 43 mg (88% isolated yield).

**^1^H NMR** (400 MHz, CDCl_3_): δ 7.58-7.52 (m, 2H), 7.51-7.41 (m, 4H), 7.34-7.29 (m, 2H), 2.64 (s, 3H).

**^13^C NMR** (101 MHz, CDCl_3_): δ 146.1, 143.2, 138.8, 132.3, 129.0, 128.9, 128.7, 128.6, 127.5, 117.8, 112.0, 21.3.

**GCMS** (EI): 95 (11), 165 (33), 192 (91), 193 (100) [M]^+^; *t_R_* = 18.24 min.

**HRMS** (ESI): *m/z* for C_14_H_12_N^+^ [M+H]^+^ calcd. 194.0964, found 194.0971.

**2-([1,1'-biphenyl]-4-yl)acetic acid (3h)**

From *p*-bromophenylacetic acid and phenylboronic acid.

White crystals, 29 mg (49% isolated yield).

**^1^H NMR** (400 MHz, D_2_O): δ 7.57-7.51 (m, 2H), 7.51-7.46 (m, 2H), 7.40-7.33 (m, 2H), 7.31-7.23 (m, 3H), 3.48 (s, 2H).

**^13^C NMR** (101 MHz, D_2_O): δ 180.8, 140.2, 138.4, 136.6, 129.7, 129.0, 127.4, 126.9, 126.7, 44.1.

**GCMS** (EI) of the methyl ester (obtained by treatment of the sample with TMS-diazomethane): 152 (11), 167 (100), 226 (42) [M]^+^; *t_R_* = 20.78 min.

**HRMS** (ESI): *m/z* for C_14_H_11_O_2_^–^ [M–H]^–^ calcd. 211.0765, found 211.0784.

**2-(2'-methoxy-[1,1'-biphenyl]-4-yl)acetic acid (3i)**

From *p*-bromophenylacetic acid and *o*-methoxyphenylboronic acid.

Pale yellow crystals, 38 mg (71% isolated yield).

**^1^H NMR** (500 MHz, CDCl_3_): δ 7.52 (d, *J*=8.2, 2H), 7.37-7.30 (m, 4H), 7.06-7.01 (m, 1H), 7.01-6.97 (m, 1H), 3.82 (s, 3H), 3.70 (s, 2H).

**^13^C NMR** (126 MHz, CDCl_3_): δ 177.9, 156.6, 137.8, 131.9, 130.9, 130.3, 129.9, 129.1, 128.8, 121.0, 111.3, 55.6, 40.9.

**GCMS** (EI) of the methyl ester (obtained by treatment of the sample with TMS-diazomethane): 152 (10), 181 (44), 197 (100), 256 (65) [M]^+^; *t_R_* = 22.25 min.

**HRMS** (ESI): *m/z* for C_15_H_13_O_3_^–^ [M–H]^–^ calcd. 241.0870, found 241.0883.

**4-hydroxy-[1,1'-biphenyl]-3-carboxylic acid (3j)**

From 5-bromosalicylic acid and phenylboronic acid.

White crystals, 29 mg (50% isolated yield).

**^1^H NMR** (500 MHz, DMSO-*d*_6_): δ 8.04 (d, *J*=2.5, 1H), 7.83 (dd, *J*=8.6, 2.5, 1H), 7.65-7.59 (m, 2H), 7.48-7.42 (m, 2H), 7.37-7.31 (m, 1H), 7.07 (d, *J*=8.6, 1H).

**^13^C NMR** (126 MHz, DMSO-*d*_6_): δ 171.8, 160.6, 139.0, 133.9, 131.3, 129.0, 128.0, 127.1, 126.2, 117.8, 113.3.

**GCMS** (EI) of the methyl ester (obtained by treatment of the sample with TMS-diazomethane): 139 (39), 168 (25), 196 (100), 228 (51) [M]^+^; *t_R_* = 20.97 min.

**HRMS** (ESI): *m/z* for C_13_H_9_O_3_^–^ [M–H]^–^ calcd. 213.0557, found 213.0565.

**4'-fluoro-4-hydroxy-[1,1'-biphenyl]-3-carboxylic acid (3k)**

From 5-bromosalicylic acid and *p*-fluorophenylboronic acid.

White crystals, 45 mg (63% isolated yield).

**^1^H NMR** (400 MHz, DMSO-*d*_6_): δ 7.99 (d, *J*=2.0, 1H), 7.80 (dd, *J*=8.7, 2.0, 1H), 7.69-7.59 (m, 2H), 7.32-7.19 (m, 2H), 7.05 (d, *J*=8.6, 1H).

**^13^C NMR** (101 MHz, DMSO-*d*_6_): δ 171.7, 161.6 (d, *J*=244.2), 160.4, 153.5 (d, *J*=3.0), 133.9, 130.3, 128.2 (d, *J*=8.2), 127.9, 117.8, 115.7 (d, *J*=21.1), 113.4.

**HRMS** (ESI): *m/z* for C_13_H_8_FO_3_^–^ [M–H]^–^ calcd. 231.0463, found 231.0471.

**4,4''-dimethyl-[1,1':3',1''-terphenyl]-5'-carbaldehyde (3l)**

****From 3,5-dibromobenzaldehyde and *p*-tolylboronic acid (2× equiv.).

Pale yellow solid, 55 mg (77% isolated yield).

**^1^H NMR** (400 MHz, CDCl_3_): δ 10.14 (s, H), 8.04 (s, 2H+1H), 7.58 (d, *J*=7.9, 4H), 7.3 (d, *J*=7.9, 4H), 2.43 (s, 6H).

**^13^C NMR** (101 MHz, CDCl_3_): δ 192.6, 142.8, 138.1, 137.5, 137.0, 131.6, 129.9, 127.2, 126.8, 21.3.

**GCMS** (EI): 242 (18), 257 (13), 286 (100) [M+1]^+^; *t_R_* = 19.51 min.

**HRMS** (ESI): *m/z* for C_19_H_15_O^+^ [M+H]^+^ calcd. 287.1430, found 287.1433.

**Copies of NMR and HRMS spectra**

**1-([1,1'-biphenyl]-4-yl)ethan-1-one (3a)**

**
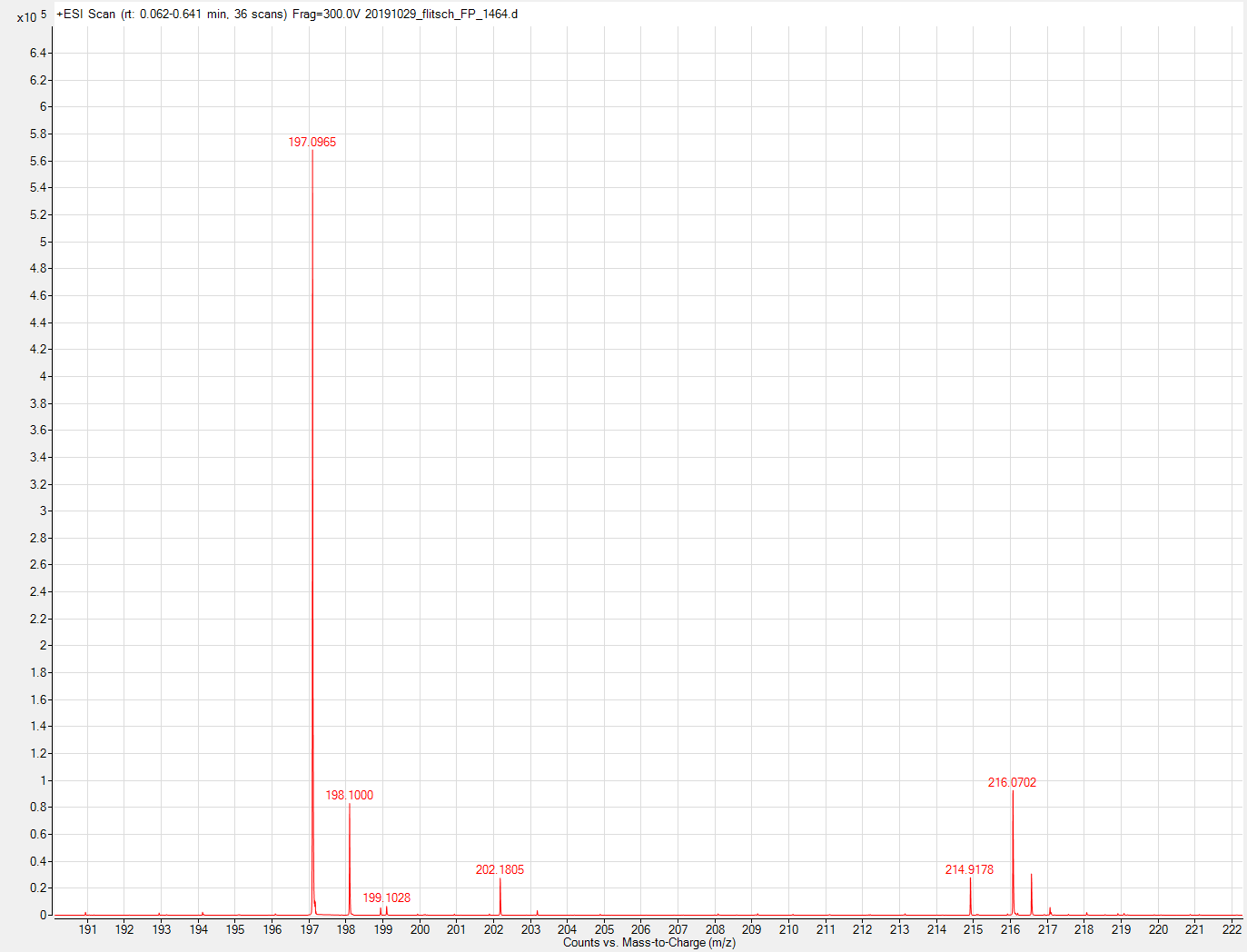
**

**1-(4'-fluoro-[1,1'-biphenyl]-4-yl)ethan-1-one (3b)**

**
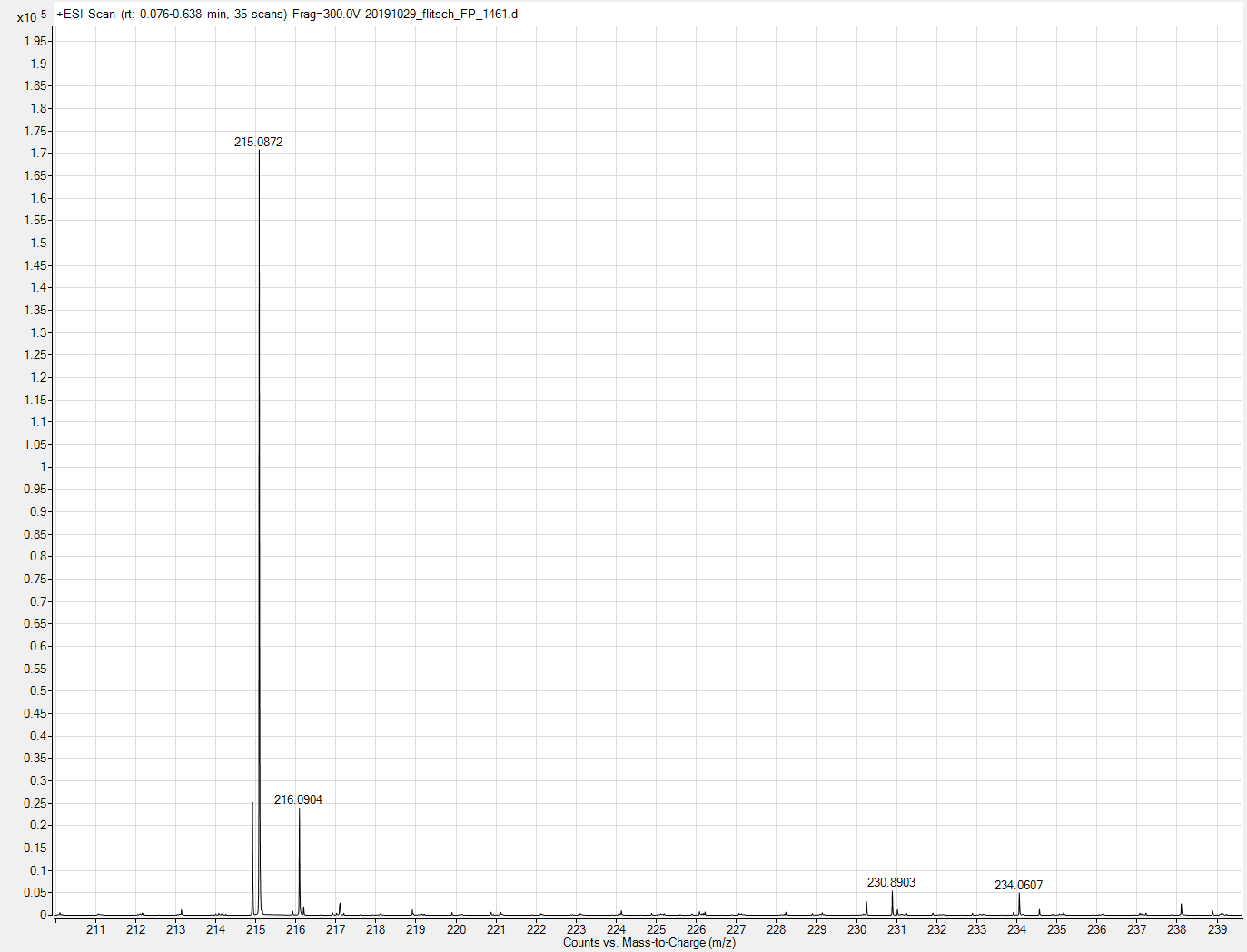
**

**1-(3'-fluoro-[1,1'-biphenyl]-4-yl)ethan-1-one (3c)**

**
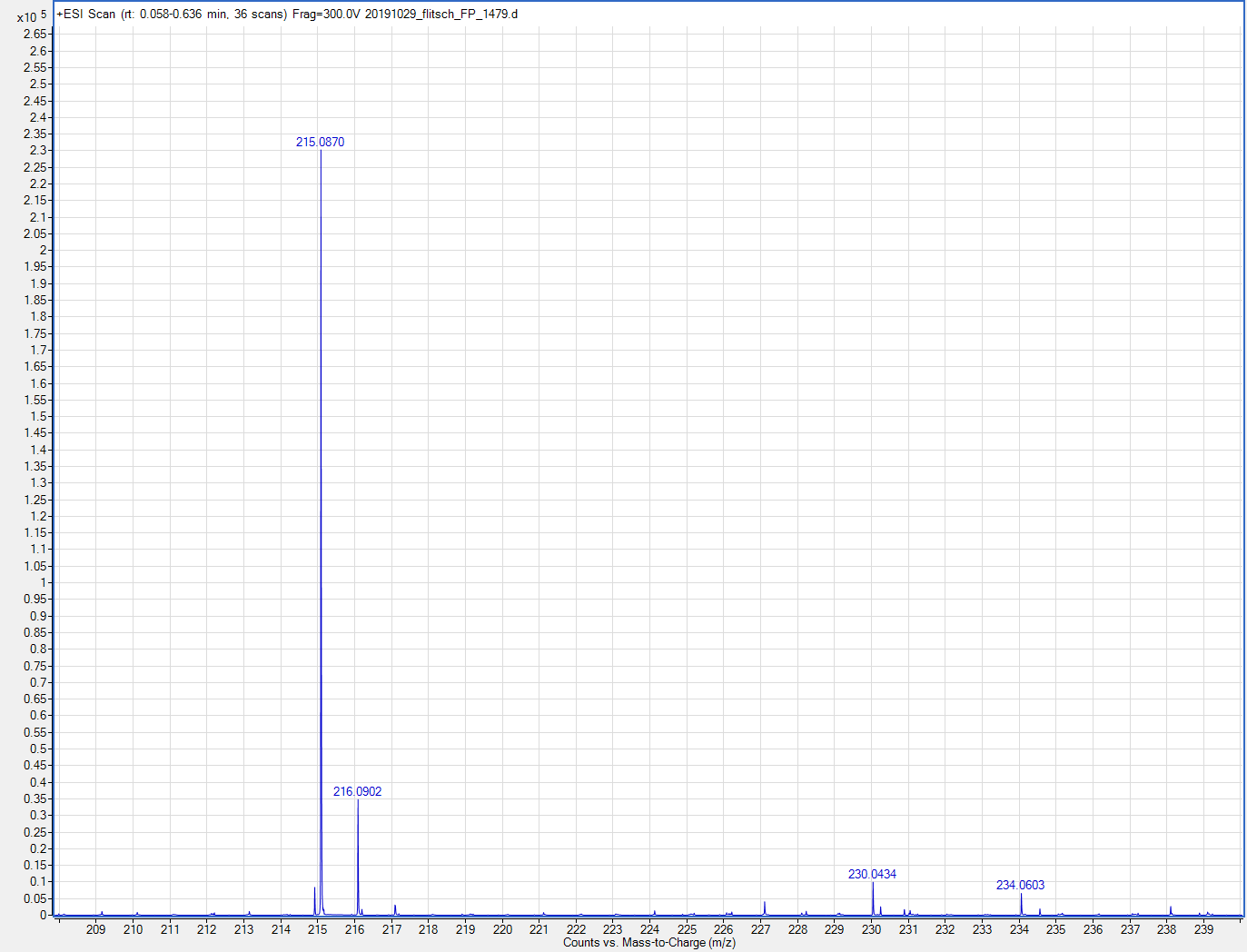
**

**1-(4'-methyl-[1,1'-biphenyl]-4-yl)ethan-1-one (3d)**

**
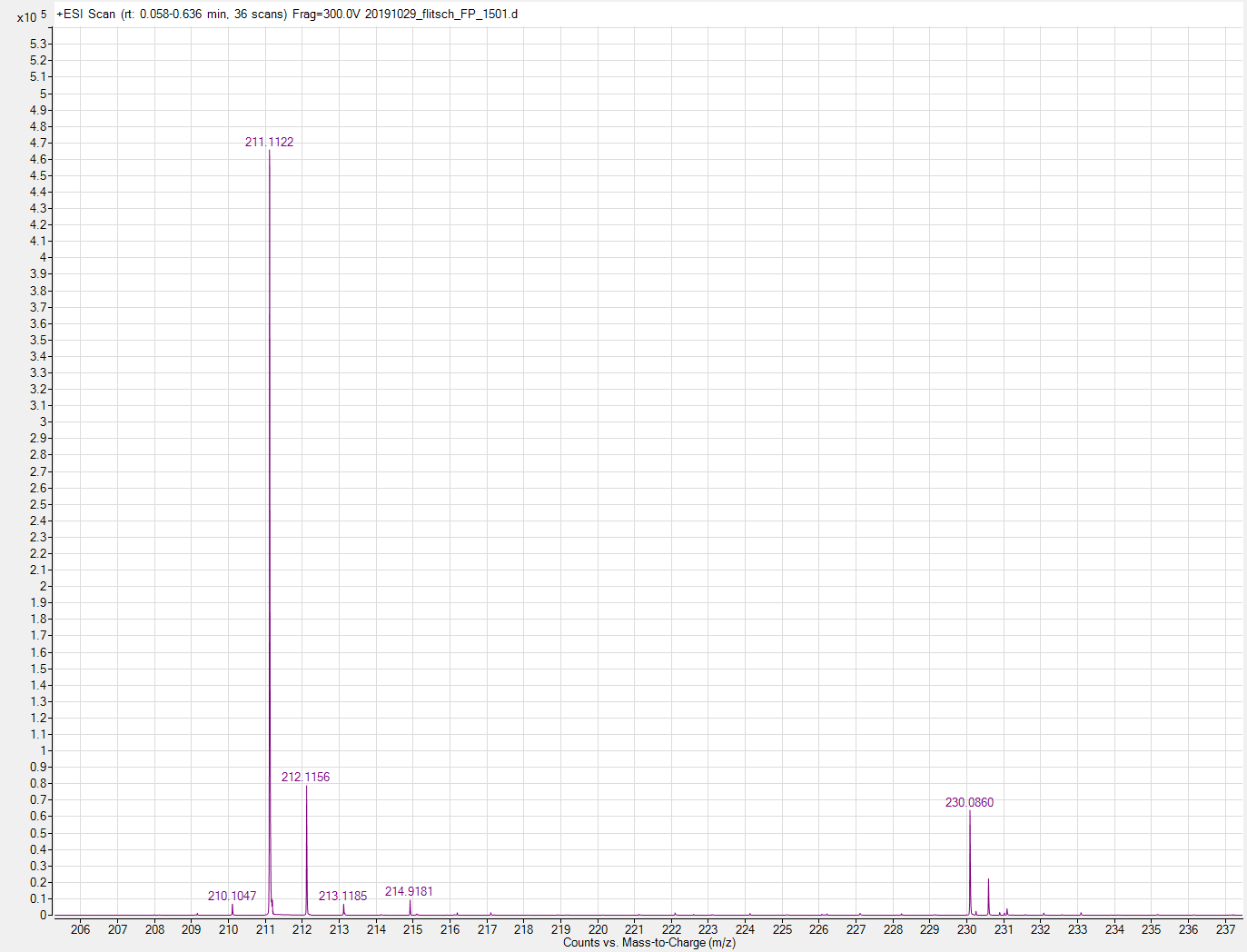
**

**1-(2'-methoxy-[1,1'-biphenyl]-4-yl)ethan-1-one (3e)**

**
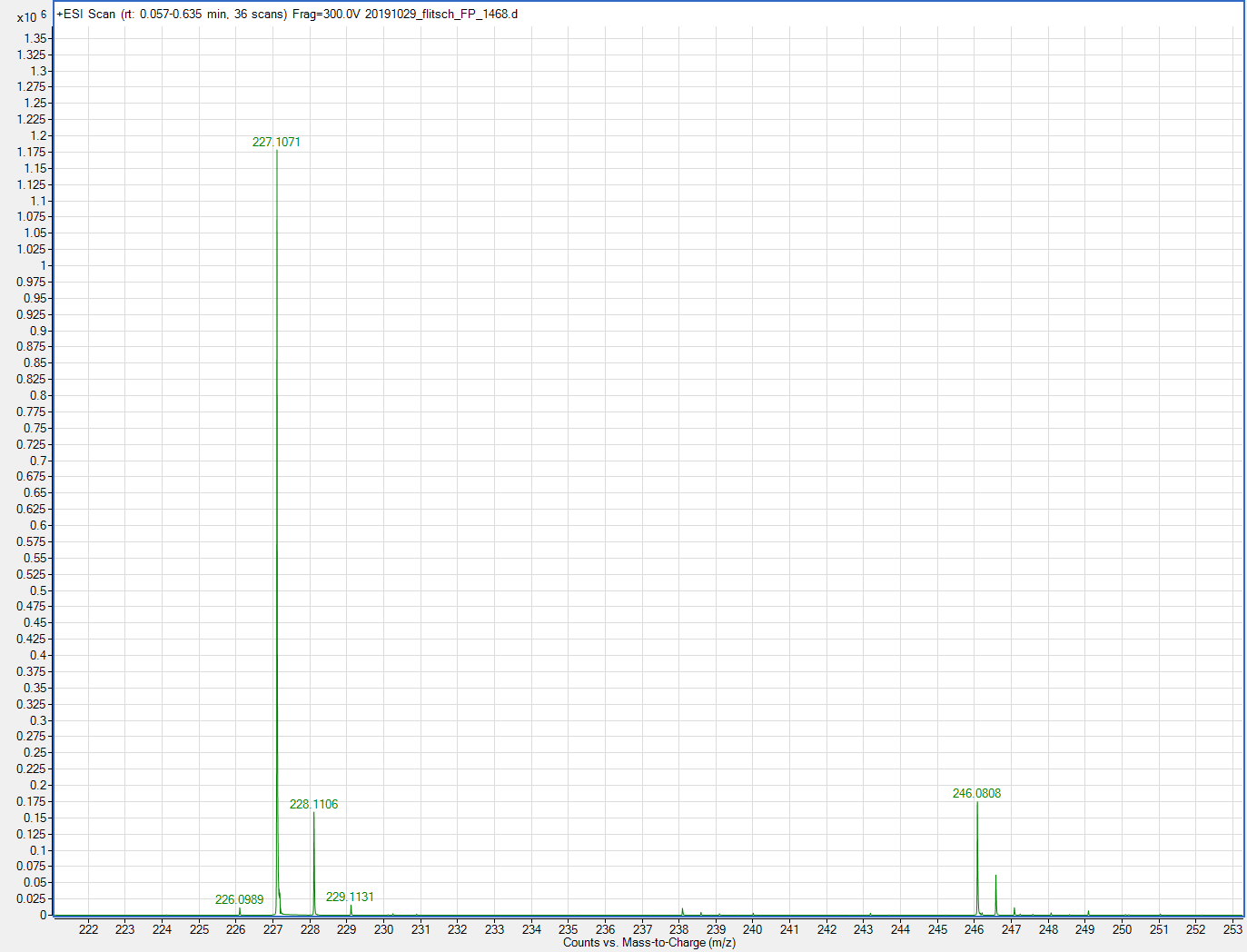
**

**1,1'-biphenyl (3f)**

**3-methyl-[1,1'-biphenyl]-2-carbonitrile (3g)**

**
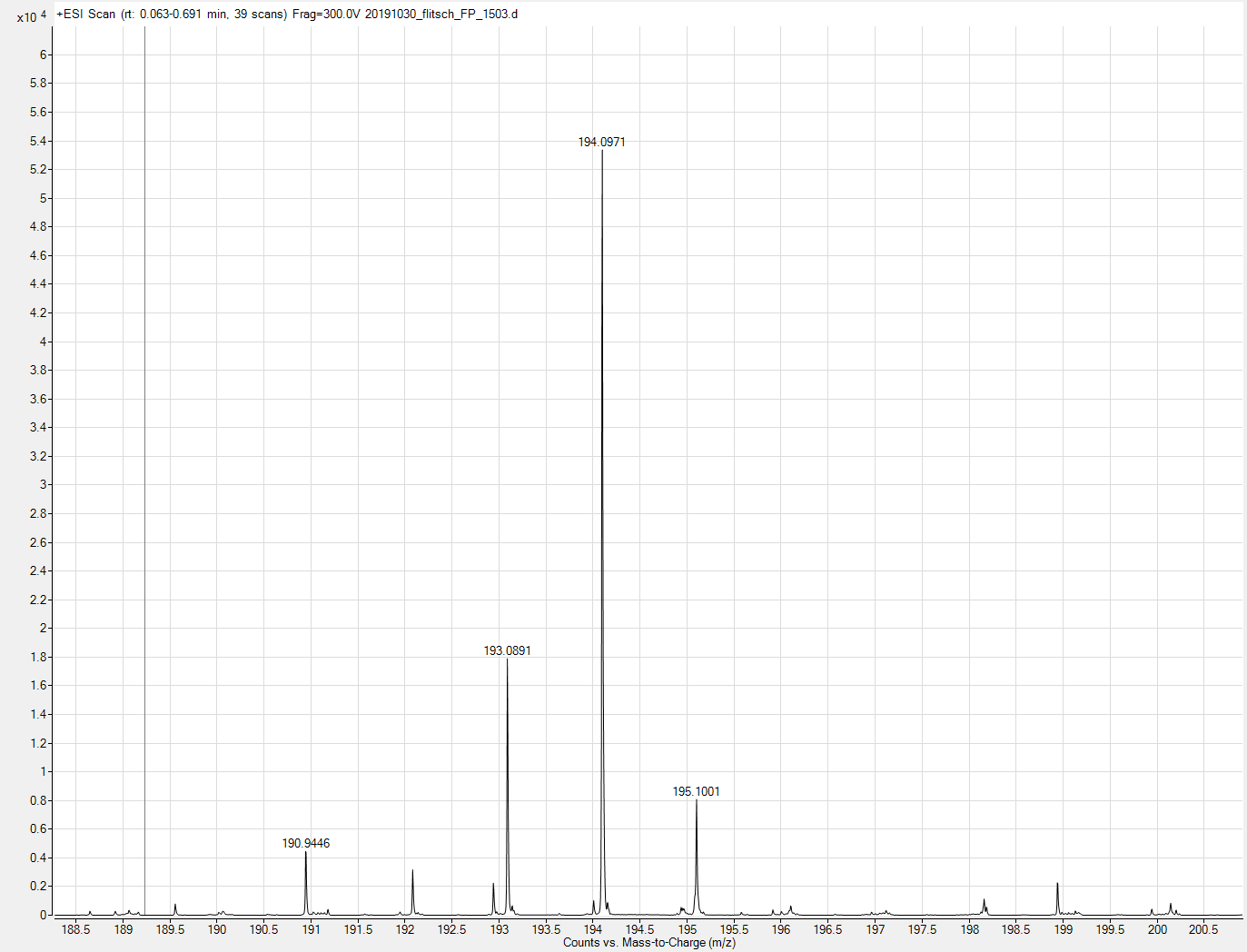
**

**2-([1,1'-biphenyl]-4-yl)acetic acid (3h)**

**
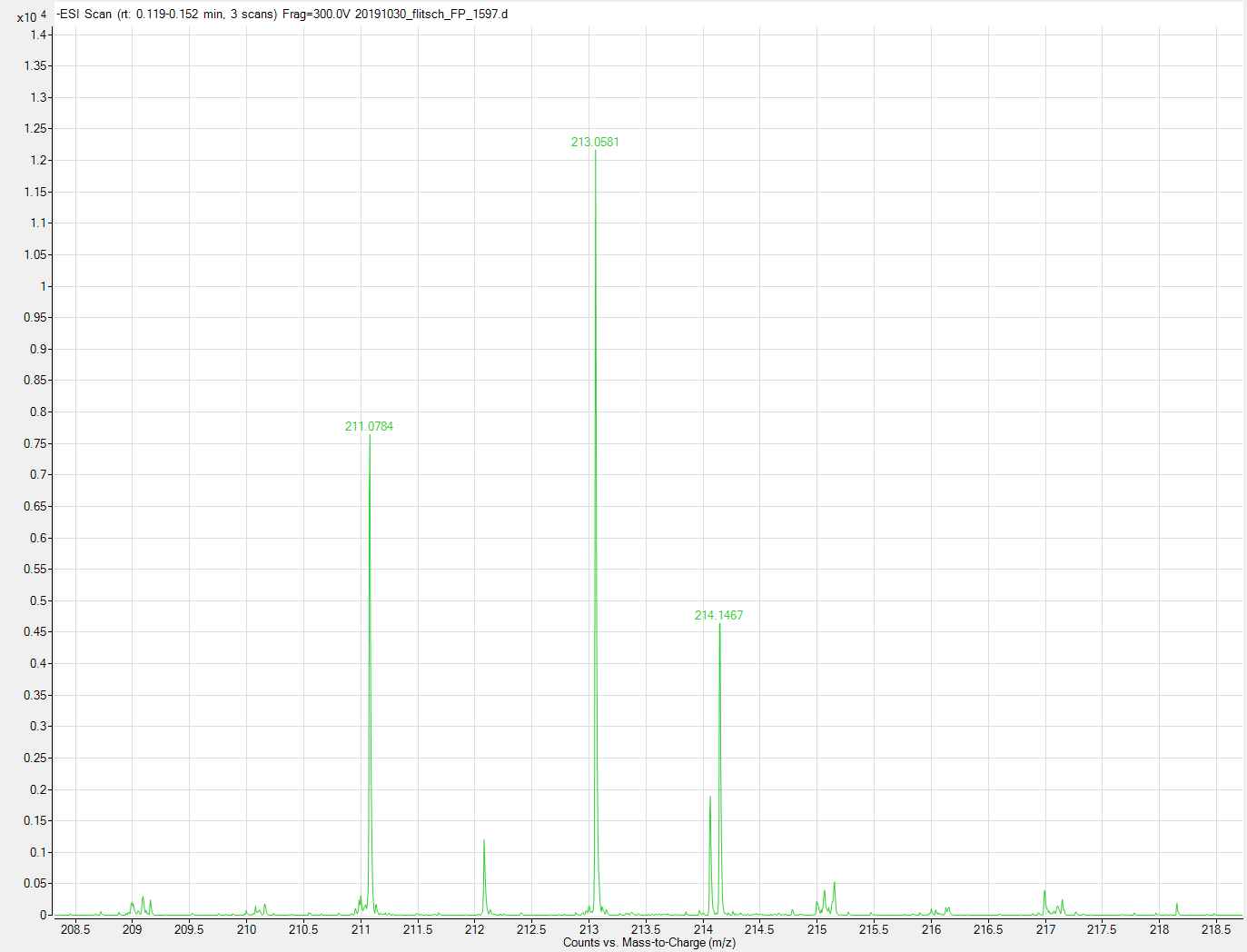
**

**2-(2'-methoxy-[1,1'-biphenyl]-4-yl)acetic acid (3i)**

**
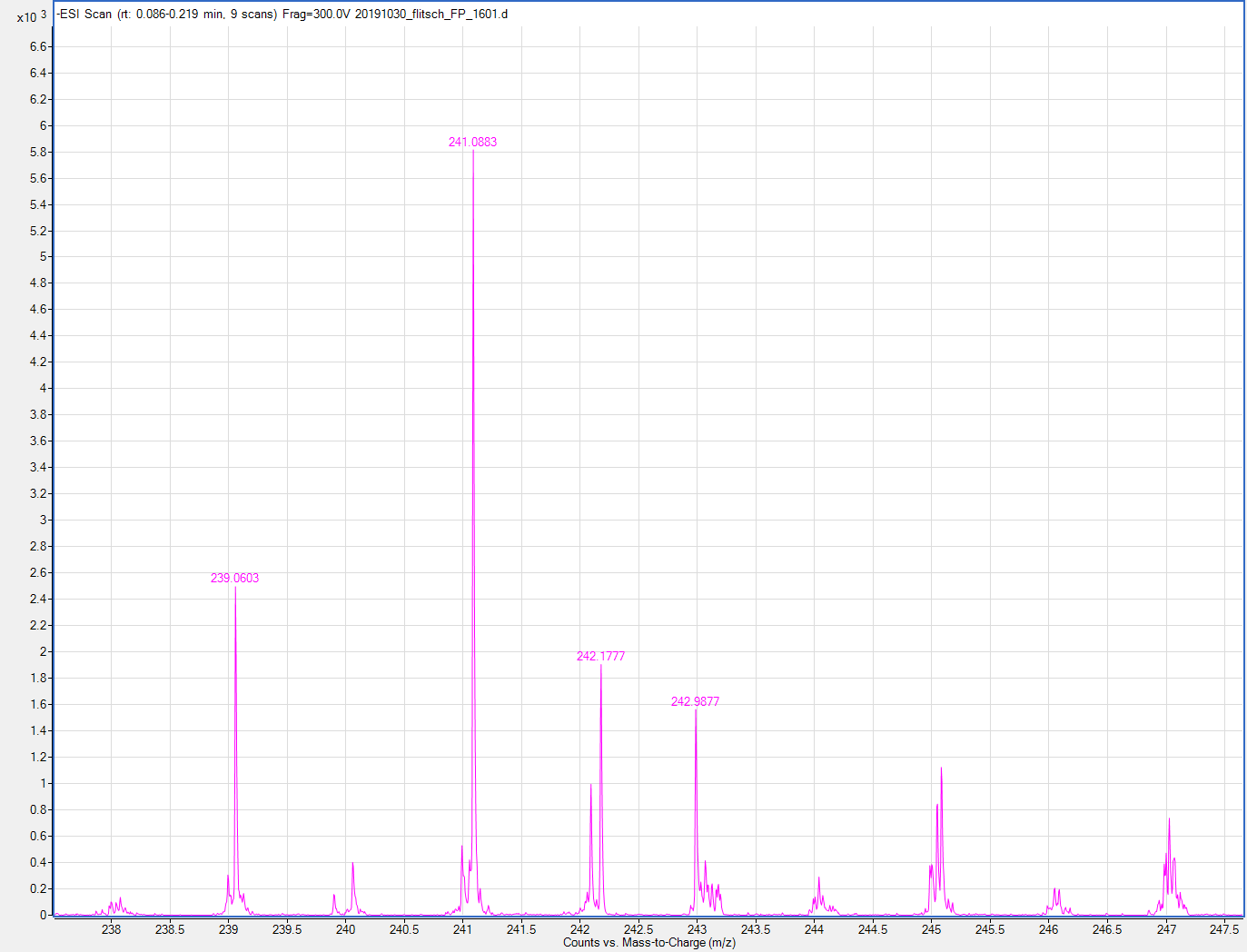
**

**4-hydroxy-[1,1'-biphenyl]-3-carboxylic acid (3j)**

**
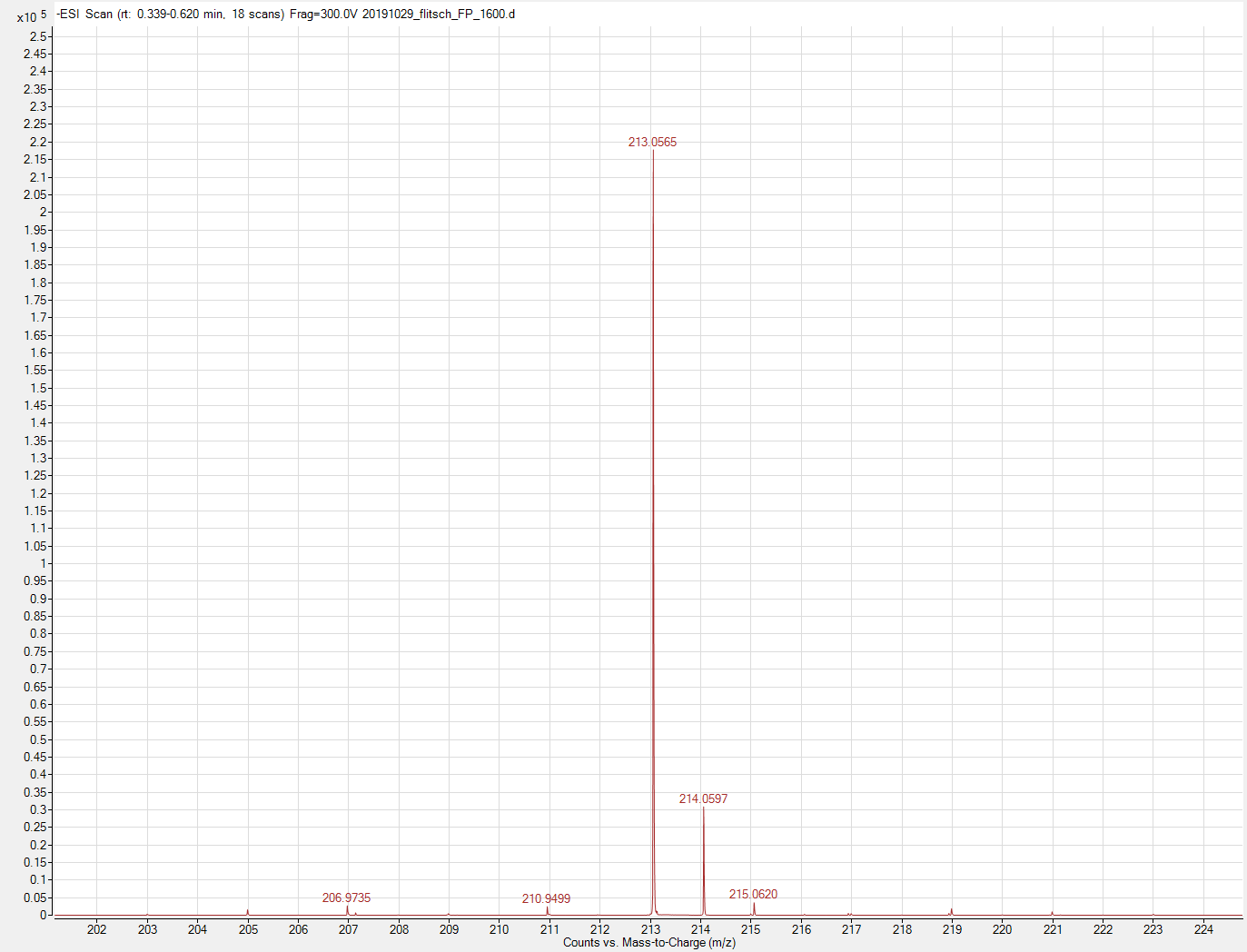
**

**4'-fluoro-4-hydroxy-[1,1'-biphenyl]-3-carboxylic acid (3k)**

**
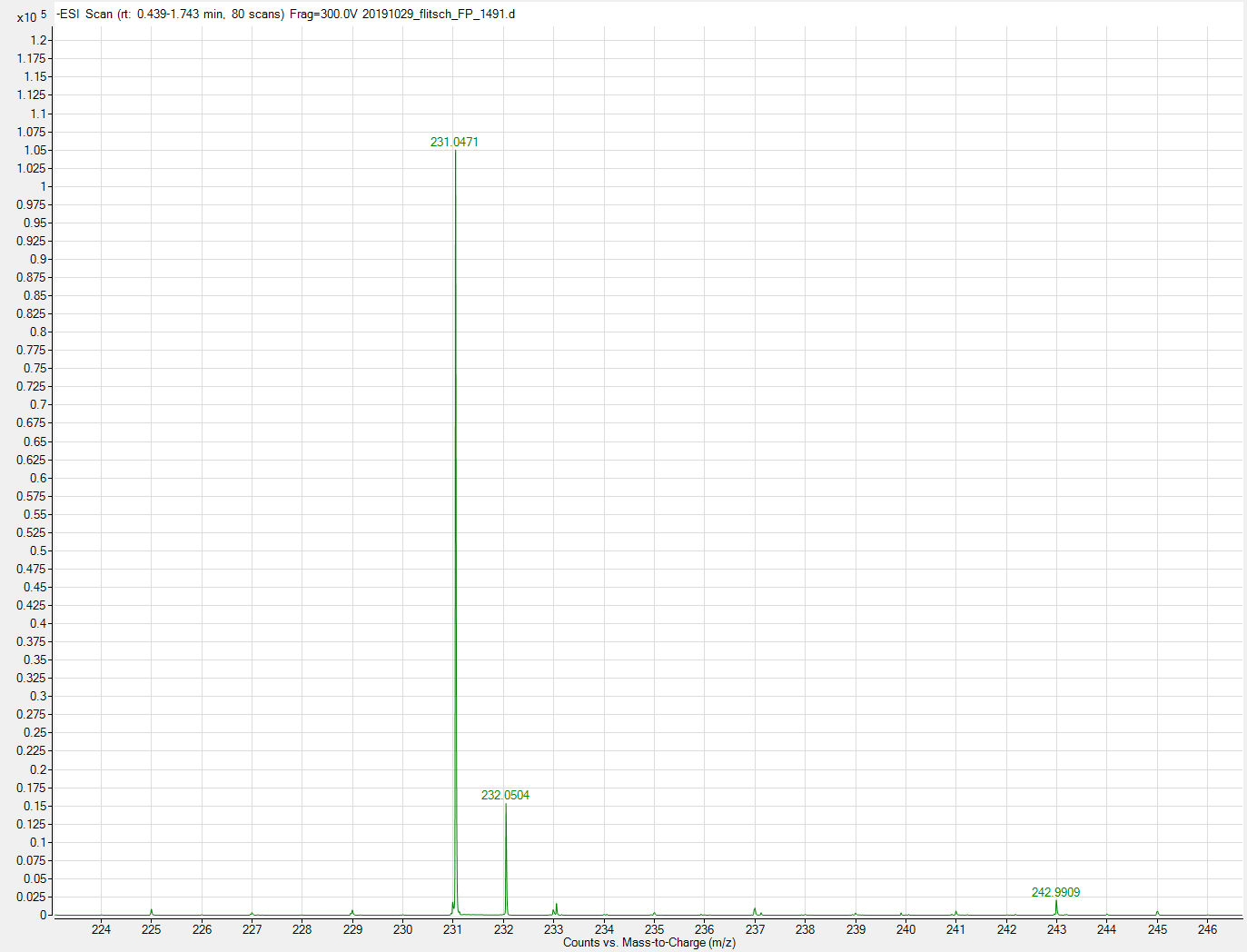
**

**4,4''-dimethyl-[1,1':3',1''-terphenyl]-5'-carbaldehyde (3l)**

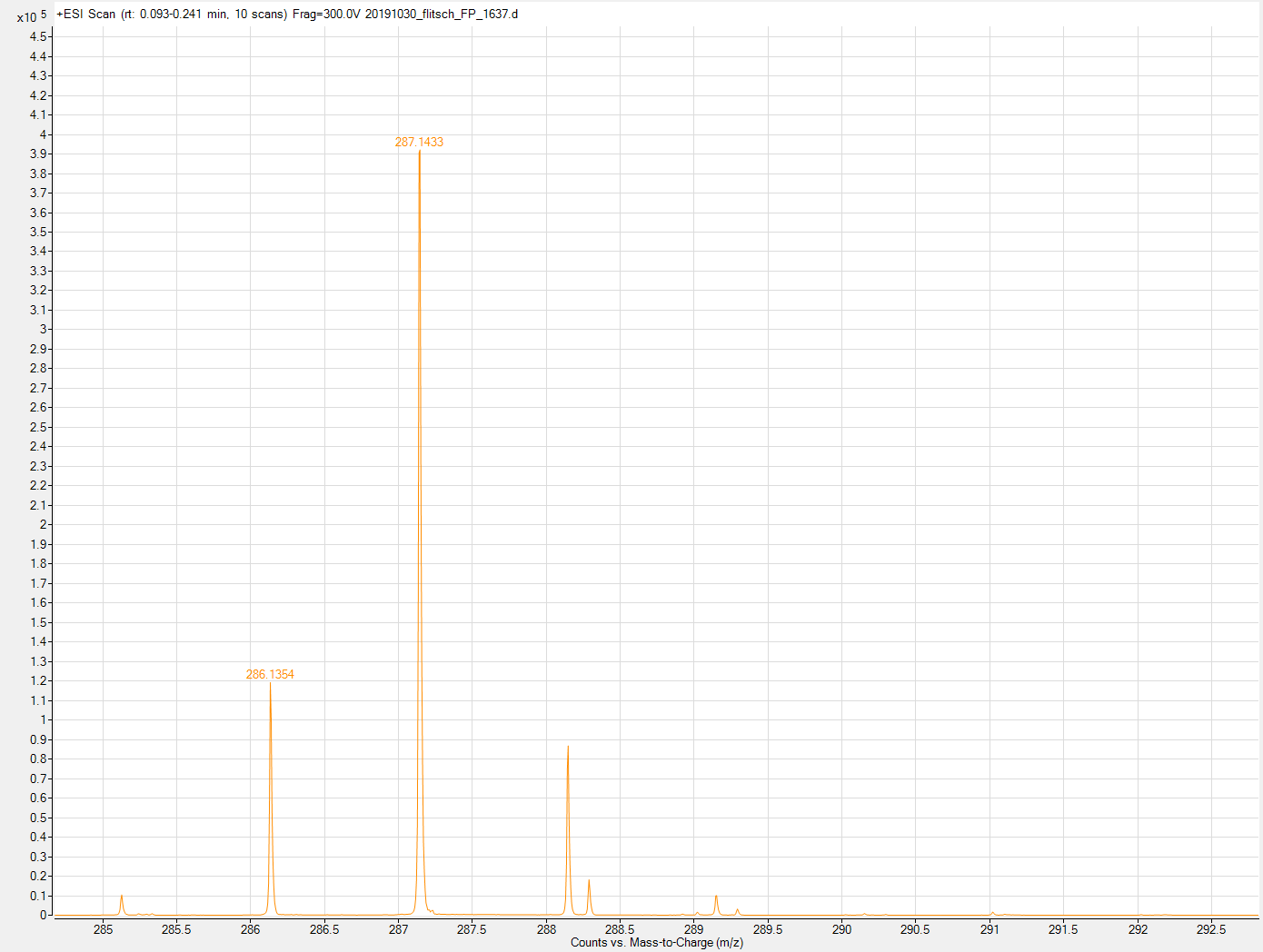

Supplement: Supplementary file 1 — Fig. S1. TEM and STEM images of Shewanella oneidensis cells after being challenged with (a‐c) no metals; (d‐e) Pd/Ag; and (f‐h) Pd/Au. Fig. S2. HAADF‐STEM image and EDX spectra of selected nanoparticles synthesised from Pd and Ag bearing‐solution. Fig. S3. HAADF‐STEM image and EDX spectra of selected nanoparticles synthesised from Pd and Au bearing‐solution. Fig. S4. HAADF and EDX STEM images of nanoparticles synthesised from Pd and Ag bearing‐solution. Fig. S5. HAADF and EDX STEM images of nanoparticles synthesised from Pd and Au bearing‐solution. Fig. S6. HAADF STEM images of nanoparticles synthesised from Pd and Ag bearing‐solution highlighting the formation of hollow nanoparticle spheres. Table S1. EXAFS fitting parameters for Pd‐K edge EXAFS of the Pd, PdAu and PdAg bioprecipitates. Coordination numbers (N), U bond distances (R (Å)), shift in energy from calculated Fermi level (ΔE0), Debye−Waller factors (σ2), amplitude reduction factors (S0) and “goodness of fit” factor (R). Coordination numbers were fixed. Numbers in parentheses are the standard deviation on the last decimal place. Table S2. EXAFS fitting parameters for Pd‐K edge EXAFS of the Pd, Pd Au and PdAg bioprecipitates including a P shell. Coordination numbers (N), U bond distances (R (Å)), shift in energy from calculated Fermi level (ΔE0), Debye−Waller factors (σ2), amplitude reduction factors (S0) and “goodness of fit” factor (R). Coordination numbers were fixed. Numbers in parentheses are the standard deviation on the last decimal place. Table S3. EXAFS fitting parameters for Ag K and Au L3 edge EXAFS of the Pd Au and PdAg bioprecipitates. Coordination numbers (N), U bond distances (R (Å)), shift in energy from calculated Fermi level (ΔE0), Debye−Waller factors (σ2), amplitude reduction factors (S0) and “goodness of fit” factor (R). Coordination numbers were fixed. Numbers in parentheses are the standard deviation on the last decimal place. Table S4. Conversions for the preparative‐scale Suzuki–Miy [file MBT2-14-2435-s001.docx]
